# Supplementary material for: Value-free random exploration is linked to impulsivity
Source: Nat Commun. 2022 Aug 4;13:4542. doi: 10.1038/s41467-022-31918-9 (PMC9352791; doi:10.1038/s41467-022-31918-9)
Supplement: Supplementary file 1 — Supplementary Information [file 41467_2022_31918_MOESM1_ESM.pdf]

Supplementary Information for

Value-free random exploration is linked to impulsivity

Magda Dubois[1,2]* and Tobias U Hauser[1,2]

[1] Max Planck UCL Centre for Computational Psychiatry and Ageing Research, London, UK
[2] Wellcome Centre for Human Neuroimaging, University College London, London, UK

* Corresponding author: Magda Dubois (magda.dubois.18@ucl.ac.uk).

Overview

Main experiment

- Supplementary Methods main experiment …………………….…………..……………… page 3
- Supplementary Note 1: Main experiment ………………………………………….…….… page 6
- Supplementary tables and figures main experiment ……………..………….……… page 9

Pilot experiment

- Supplementary Note 2: Pilot experiment …………………………………………… page 38
- Supplementary tables and figures pilot experiment …………………………… page 41

Supplementary References ……………………………………………………….……..…………… page 58

Supplementary Methods main experiment

Model descriptions

In this study, we use the models that were developed and validated for this task in our previous work[1]. Here, we re-print these equations for completion. For a summary of the parameters of each model cf. Supplementary Table 4. The value of each bandit is represented as a distribution $N(Q,S)$ with $S = 0.8$. Participants have prior beliefs about bandits' values which we assume to be Gaussian with mean Q_0 (prior mean; free parameter) and uncertainty σ_0 (prior variance; free parameter).

Mean and variance update rules

At each time point t, in which a sample m, of one of the bandits is presented, the expected mean Q and precision $\tau = \frac{1}{\sigma^2}$ of each bandit are updated as follows:

$$Q_{i,t+1} = \frac{\tau_{i,t} * Q_{i,t} + \tau_{samp} * m}{\tau_{i,t} + \tau_{samp}} \quad (1)$$

$$\tau_{t+1}^{i} = \tau_{samp} + \tau_{t}^{i} \quad (2)$$

with $\tau_{samp} = \frac{1}{S^2}$ the sampling precision, $S = 0.8$ the fixed sampling variance, m the presented sample, i is the bandit and t the time point. Those update rules are equivalent to using a Kalman filter[2] in stationary bandits.

Models

We examined three base models reflecting complex exploration strategies: the UCB model, the Thompson model and the hybrid model. The UCB model encompasses the UCB algorithm (captures directed exploration) and a softmax choice function (captures the so-called value-based random exploration). The Thompson model reflects Thompson sampling (captures an uncertainty-dependent exploration). The hybrid model captures the contribution of the UCB model and the Thompson model, essentially a mixture of the above. We will compute three extensions of each model by either adding value-free random exploration (c_{vf},c_n)={1,0}, novelty exploration (c_{vf},c_n)={0,1} or both heuristics (c_{vf},c_n)={1,1}. To make sure that the UCB models are not penalized because of the higher number of free parameters compared to the Thompson sampling models, we will also look at the UCB models with fixed inverse temperature parameter (β=1). This leads to a total of 16 models (see the labels on the x-axis in Supplementary Figure 5). A coefficient c_{vf}=1 indicates that a ϵ-greedy component was added to the decision rule, ensuring that once in a while (every ϵ % of the time), another option than the predicted one is selected. A coefficient c_n=1 indicates that the novelty bonus η is added to the computation of the value of novel bandits and the Kronecker delta δ in front of this bonus ensures that it is only applied to the novel bandit. For each model, the probability of choosing a bandit is described below. Please note that these three complex models make relatively similar predictions in our task, and that our model selection is primarily targeted at establishing the presence of exploration heuristics (in addition to complex exploration strategies).

UCB model:

In this model, an information bonus γ is added to the expected mean Q of each option, scaling with the option's uncertainty σ.

$$P(i) = \frac{e^{\beta V_{i,t}}}{\sum_x e^{\beta V_{i,t}}} * (1 - c_{vf}\epsilon) + c_{vf}\frac{\epsilon}{3}$$

(3)

$$\text{with } V_{i,t} = Q_{i,t} + \gamma\sigma_{i,t} + c_n\eta\delta_{[i=novel]}$$

γ the information bonus, V the expected value, η the novelty bonus and β the inverse temperature of the softmax (lower values producing more stochasticity). A UCB model with a fixed inverse temperature (model: UCB (β=1) in Supplementary Figure 5) was also included.

Thompson sampling model:

In this model, the overall uncertainty can be seen as a more refined version of a decision temperature[3]. At each time step, a sample $x_{i,t} \sim \mathcal{N}(V_{i,t}, \sigma_{i,t}^2)$ is taken from each bandit. The probability of choosing a bandit i depends on the probability that all pairwise differences (contained in the two-dimensional vector u) between the sample from bandit i and the other bandits $j \neq i$ were greater or equal to 0.

$$P(i) = P(\forall j: x_{i,t} > x_{j,t}) * (1 - c_{vf}\epsilon) + c_{vf}\frac{\epsilon}{3}$$
$$= \int_0^\infty \int_0^\infty \phi(u; M_{i,t}, C_{i,t})\, du\ * (1 - c_{vf}\epsilon) + c_{vf}\frac{\epsilon}{3}$$

(4)

$$\text{with } M_{i,t} = A_i \begin{pmatrix} V_{1,t} \\ V_{2,t} \\ V_{3,t} \end{pmatrix}, \quad C_{i,t} = A_i \begin{pmatrix} \sigma_{1,t} & 0 & 0 \\ 0 & \sigma_{2,t} & 0 \\ 0 & 0 & \sigma_{3,t} \end{pmatrix} A_i^T,$$
$$V_{i,t} = Q_{i,t} + c_n\eta\delta_{[i=novel]},$$

ϕ the multivariate Normal density function, A the matrix computing the pairwise differences for each bandit and $x_{i,t} \sim \mathcal{N}(V_{i,t}, \sigma_{i,t}^2)$ a sample taken from each bandit.

Hybrid model:

This model allows a combination of the UCB model and the Thompson model[3].

$$P(i) = \left(wP_{UCB}(i) + (1 - w)P_{Thompson}(i)\right) * (1 - c_{vf}\epsilon) + c_{vf}\frac{\epsilon}{3}$$

(5)

with w the contribution of the UCB and Thompson sampling models

Parameter estimation

To fit the parameter values, we used the maximum a posteriori probability (MAP) estimate. The optimisation function used is fmincon in MATLAB. All the parameters besides Q_0 and w were free to vary as a function of the horizon as they capture different exploration forms: directed exploration (information bonus γ; UCB model), novelty exploration (novelty bonus η), random exploration (inverse temperature β; UCB model), uncertainty-directed exploration (prior variance σ_0; Thompson model) and value-free random exploration (ϵ-greedy parameter). The prior mean Q_0 was fitted to both horizons together as we did not expect the belief of how good a bandit is to depend on the horizon. The same holds for w, as we assume that the arbitration between the UCB model and the Thompson model does not depend on the horizon. The parameters were permitted to vary within the following bounds: $\sigma_0 = [0.01, 6]$, $Q_0 = [1, 10]$, $\epsilon = [0, 0.5]$, $\eta = [0, 5], \gamma = [0, 0.5]$, $\beta = [1.4, 5]$, $w = [0, 1]$. The prior distribution used for the prior mean Q_0 was the normal distribution that approximate the generative distributions: $Q_0 \sim \mathcal{N}(5, 2)$. For the ϵ-greedy parameter, the novelty bonus η and the prior variance σ_0, a uniform distribution was used (equivalent to performing maximum likelihood estimation). We have found that the model parameters are well recoverable in our current (Supplementary Figure 6, Supplementary Figure 19 and Supplementary Figure 20) and previous simulation analyses[1].

Model comparison

Model comparison was performed using the Bayesian Information Criterion[4] (BIC). We computed the mean BIC score across participants and the number of participants which are best fit for each model. Additionally, we used a Bayesian model framework and computed the exceedance probability of each model[5].

Parameter recovery

For each parameter of the winning model (Thompson+$\eta + \epsilon$ from the pilot data), we sampled parameter values from a normal distribution defined by the pilot data mean and standard deviation, which we used to simulate behaviour. This was performed N=1000 times. For each simulation, we fitted the model and analysed the correlation between the simulated parameters and the fitted parameters (for confusion matrix cf. Supplementary Figure 6a and Supplementary Figure 19a; for a visualisation of each parameters' correlation cf. Supplementary Figure 20). Similar results were obtained when simulating behaviour from parameter values sampled from the fitted values obtained in the pilot data (Supplementary Figure 24).

Model identification

For each model, behaviour was simulated N=100 times with parameter values sampled from the pilot data mean and standard deviation. All models were fitted to this simulated data and BIC scores compared. The percentage of how often (out of the N=100 simulations) each fitted model won was computed (i.e., confusion matrix, cf. Supplementary Figure 6b and Supplementary Figure 19b). Additionally, the inversion matrix[6] was computed (cf. Supplementary Figure 6c). Please note that key to our model comparison is to assess the benefit of the two exploration heuristics (novelty exploration, value-free random exploration). We expected a degree of trade-off between the different complex models, as they make relatively similar predictions.

Supplementary Note 1: Main experiment

The analyses mentioned below were not preregistered. The first part are analyses which were performed on pilot data in the Stage 1 report, the second part are new analyses (i.e., added after data collection).

Additional analyses (part of the Stage 1 report)

Further analysis of the high-value bandit frequency

The horizon effect on the frequency of picking the high-value bandit was independent of whether the high-value bandit had 3 initial samples (V=153594.5, p<.001, r=0.809) or 1 prior sample (V=134660, p<.001, r=0.633) associated to it (samples main effect: F(1, 579)=486.649, p<.001, η^2 =.457). Interestingly this horizon effect was stronger in the former (samples-by-horizon interaction effect: F(1, 579)=333.132, p<.001, η^2=.365; horizon main effect: F(1,579)=634.19, p<.001, η^2=.365), in line with predictions from uncertainty-guided exploration strategies.

Horizon effect on the certain-standard and standard bandits

The frequency of picking the certain-standard bandit was increased in the long versus short horizon (V=157616.5, p<.001, r=0.797; Supplementary Figure 9a). Similarly, the frequency of picking the standard bandit was increased in the long horizon (V=128795.5, p<.001, r=.543; Supplementary Figure 9b).

Block and trial effect on the high-value bandit

In addition to the horizon condition, when adding block as a within participant-factor in the repeated-measures ANOVA, a block main effect was observed (F(2.49, 1441.95)=100.789, p<.001, η^2=0.148; horizon main effect: F(1, 579)=634.19, p<.001, η^2=0.523). This was driven by a decreased selection frequency of the high-value bandit with increasing blocks (pairwise comparison: block 1 vs block 2: t(1160)=12.837, p<.001, d=0.377, 95%CI_M = [0.988,1.345]; block 1 vs block 3: t(1160)=13.266, p<.001, d=0.39, 95%CI_M = [1.072,1.445]; block 1 vs block 4: t(1160)=16.252, p<.001, d=0.477, 95%CI_M = [1.41,1.798]; block 2 vs block 3: t(1160)=1.277, p=.202, d=0.037, 95%CI_M = [-0.05,0.234]; block 2 vs block 4: t(1160)=5.784, p<.001, d=0.17, 95%CI_M = [0.289,0.586]; block 3 vs block 4: t(1160)=4.439, p<.001, d=0.13, 95%CI_M = [0.193,0.498]). Similarly, when analysing the frequency of picking the high-value bandit per trial, a decrease was observed in the short horizon (linear regression slope vs null slope: t(579)=-13.7, p<.001) and in the long horizon (t(579)=-19.3, p<.001). Additionally, the horizon effect seemed to be stronger in later blocks (block-by-horizon interaction: F(2.87, 1663.52)=6.716, p<.001, η^2=0.011; pairwise comparisons: horizon effect in: Block 1: t(580)=-17.887, p<.001, d=0.743, 95%CI_M = [-2.465,-1.978]; Block 2: t(580)=-17.623, p<.001, d=0.732, 95%CI_M = [-2.632,-2.104]; Block 3: t(580)=-20.086, p<.001, d=0.834, 95%CI_M = [-3.027,-2.488]; Block 4: t(580)=-20.545, p<.001, d=0.853, 95%CI_M = [-2.956,-2.44]).

No block nor trial effect on the low-value bandit

In addition to the horizon condition, when adding block as a within participant-factor in the repeated-measures ANOVA, there was no significant effect of block on the low-value bandit (F(2.67,1547.6)=2.571, p=.06, η^2=0.004; horizon main effect: F(1,579)=113.659, p<.001, η^2=0.164; Block-by-Horizon interaction effect: F(3,1737)=0.382, p=.766, η^2=0.001). Similarly, when analysing the frequency of picking the low-value bandit per trial, no evidence of changes across time were observed, neither in the short horizon (linear regression slope vs null slope: t(579)=-1.65, p=.099) nor in the long horizon (t(579)=-1.07, p=.287).

Block and trial effect on the novel bandit

In addition to the horizon condition, when adding block as a within participant-factor in the repeated-measures ANOVA, there was an additional effect of block on the novelty bandit (F(2.45,1416.75)=135.038, p<.001, η^2 =0.189; horizon main effect: F(1,579)=504.394, p<.001, η^2=0.466). This was driven by an increased selection frequency of the novel bandit with increasing blocks (pairwise comparison: block 1 vs block 2: t(1160)=-15.221, p<.001, d=0.447, 95%CI_M = [-1.598,-1.233]; block 1 vs block 3: t(1160)=-15.71, p<.001, d=0.461, 95%CI_M = [-1.687,-1.313]; block 1 vs block 4: t(1160)=-18.59, p<.001, d=0.546, 95%CI_M = [-2.119,-1.714]; block 2 vs block 3: t(1160)=-1.17, p=.242, d=0.034, 95%CI_M = [-0.226,0.057]; block 2 vs block 4: t(1160)=-6.263, p<.001, d=0.184, 95%CI_M = [-0.658,-0.344]; block 3 vs block 4: t(1160)=-5.271, p<.001, d=0.155, 95%CI_M = [-0.571,-0.261]). Similarly, when analysing the frequency of picking the novel bandit per trial, an increase was observed in the short horizon (linear regression slope vs null slope: t(579)=16.5, p<.001) and in the long horizon (t(579)=20.9, p<.001). Additionally, the horizon effect seemed to be milder in the 2nd block (block-by-horizon interaction: F(2.86,1654.18)=5.026, p=.002, η^2=0.009; pairwise comparisons: horizon effect in: Block 1: t(580)=17.538, p<.001, d=0.728, 95%CI = [1.918,2.401]; horizon effect in: Block 2: t(580)=15.42, p<.001, d=0.64, 95%CI_M = [1.877,2.425]; horizon effect in: Block 3: t(580)=18.435, p<.001, d=0.765, 95%CI_M = [2.353,2.914]; horizon effect in: Block 4: t(580)=17.36, p<.001, d=0.721, 95%CI_M = [2.065,2.592]).

Additional block-dependent novelty parameter

Given the observed minor change in the novelty bandit frequency across blocks and trials (cf. above), we extended our model comparison with a model comprising a block-dependent quantity of the novelty bonus, which we named η_B. This model performed similarly to our winning model when looking at the average score (Thompson+ϵ+η: BIC: 509.11 (sd: 118.81); Thompson+ϵ+η+η_B: BIC: 511.54 (sd: 120.26); paired-samples t-test: t(579)=-3.700, p<.001, 95%CI=[-3.728,-1.142]) but less well than our winning model when looking at the best score per participant (Thompson+ϵ+η: Number of participants: 181; Thompson+ϵ+η+η_B: Number of participants: 124). Importantly adding such a parameter did not affect our main parameter of interest ϵ (correlation between ϵ from the Thompson+ϵ+η model and ϵ from the Thompson+ϵ+η+η_B model: short horizon: r=0.904, p<.001; long horizon: r=.092, p<.001).

Meta-learning on number of initial samples

We found no evidence for choosing any 1-sample bandit (standard or low-value bandit) less compared to the 3-sample bandit over time (linear regression between [3-sample bandit frequency minus 1-sample bandit frequency] and [trial]: slope beta vs null slope: t(579)=-1.48, p=.139, beta=-0.005). There was a mild decrease in certain-standard (versus standard) bandit selection over time (linear regression between [certain-standard bandit frequency minus standard bandit frequency] and [trial]: slope vs null slope: t(579)=-2.07, p=.039, beta=-0.007) in the long horizon (t(579)=-3.33, p<.001; short horizon: t(579)=0.1, p=.92).

No difference in bandit colour occurrence

When performing an ANOVA with the participant identifier colour (8 sets of 3 different colours, 8^3 =24 different colours) and the within factor bandit there was no evidence of a difference in bandit occurrence for each colour (bandit main effect: F(1.8,41.47)=0, p=1, η^2=0), meaning that each type of bandit is shown in every combination equally often.

Additional analyses (not part of the Stage 1 report)

No block and trial effect on 1st samples' score

For each participant, we performed a regression on the average reward per block as well as a regression on the reward per trial. When analysing the 1st sample's reward per block, no evidence of changes across time were observed, neither in the short horizon (linear regression slope vs null slope: $t(579)=0.249$, $p=0.804$; Supplementary Figure 4a) nor in the long horizon ($t(579)=0.104$, $p=0.917$; Supplementary Figure 4b). Similarly, when analysing it per trial, no effect was observed, neither in the short horizon ($t(579)=-0.153$, $p=0.878$; Supplementary Figure 4d), nor in the long horizon ($t(579)=0.156$, $p=0.876$; Supplementary Figure 4e). When analysing the full reward in the long horizon (i.e., sum of 6 samples), a mild increase over time was observed (over blocks: $t(579)=5.409$, $p<0.001$; Supplementary Figure 4c; over trials: $t(579)=5.24$, $p<0.001$; Supplementary Figure 4f). Importantly, there is no evidence that performance changed across time on the data used for the main analysis (1st samples only).

Supplementary tables and figures main experiment

Measure	Mean (std)	Range
N=580 (304 female)	-	-
Age	35.7 (13.6)	[18, 83]
ICAR sample test (IQ)	88.7% (33.6%)	[10, 160]
BIS (impulsivity)	60.4 (10.1)	[35, 101]
ASRS (ADHD)	44.5 (11.6)	[18, 88]
AQ10 (autism)	3.31 (2.15)	[0, 10]
CFS (cognitive flexibility)	52.6 (7.54)	[29, 72]
OCIR (OCD)	15.9 (12.4)	[0, 67]
STAI (trait anxiety)	45.2 (12.6)	[20, 78]
IUS (uncertainty intolerance)	68.7 (22.5)	[28, 135]
SDS (depression)	41.2 (10.4)	[20, 70]
LSAS (social anxiety)	52.3 (27.1)	[0, 142]

Supplementary Table 1. Participant's demographics. Information about participant's gender, age and questionnaire scores. The IQ score was computed as the sum of the correct answers on the ICAR sample test[9]. The mean scores of the BIS, ASRS, AQ10, CFS, OCIR, STAI, IUS, SDS and LSAS questionnaires are the total scores.

Measure	Shapiro normality assumption	Difference of the mean (t-test)	Wilcoxon signed-rank test
Expected value	SH: p<.001<br>LH: p<.001	t(579)=7.257, p<.001,<br>95%CI_m=[0.057,0.1], d=-0.301,<br>95%CI_d=[-0.385,-0.218]	V=110057,<br>p<.001,<br>r=.265
Chosen bandits' initial samples	SH: p<.001<br>LH: p<.001	t(579)=25.298, p<.001,<br>95%CI_m=[0.224,0.262], d=-1.05,<br>95%CI_d=[-1.153,-0.95]	V=160109.5,<br>p<.001,<br>r=.796
Score 1st SH vs 1st LH	SH: p=.003<br>1stLH: p=.025	t(579)=14.3, p<.001,<br>95%CI_m=[0.057,0.075], d=-0.594,<br>95%CI_d=[-0.682,-0.506]	V=131612,<br>p<.001,<br>r=.53
Score 1st LH vs all LH	1stLH: p=.025<br>allLH: p<.001	t(579)=-51.488, p<.001,<br>95%CI_m=[-0.307,-0.284], d=2.138,<br>95%CI_d=[1.991,2.287]	V=264,<br>p<.001,<br>r=.864
Score 1st SH vs all LH	SH: p=.003<br>allLH: p<.001	t(579)=-41.711, p<.001,<br>95%CI_m=[-0.24,-0.218], d=1.732,<br>95%CI_d=[1.604,1.862]	V=1257.5,<br>p<.001,<br>r=.853
High-value bandit frequency	SH: p<.001<br>LH: p<.001	t(579)=25.183, p<.001,<br>95%CI_m=[9.262,10.829], d=-1.046,<br>95%CI_d=[-1.148,-0.945]	V=157079.5,<br>p<.001,<br>r=.797
Novel bandit frequency	SH: p=.002<br>LH: p<.001	t(579)=-22.459, p<.001,<br>95%CI_m=[-10.083,-8.462], d=0.933,<br>95%CI_d=[0.835,1.031]	V=10355,<br>p<.001,<br>r=.750
Low-value bandit frequency	SH: p<.001<br>LH: p<.001	t(579)=-10.661, p<.001,<br>95%CI_m=[-1.301,-0.896], d=0.443,<br>95%CI_d=[0.358,0.528]	V=34420,<br>p<.001,<br>r=.425
BIC: Thompson+ϵ+η vs UCB+ϵ+η	Thompson: p<.001<br>UCB: p<.001	t(579)=-8.13, p<.001,<br>95%CI_m=[-8.757,-5.349], d=-0.338,<br>95%CI_d=[-0.421,-0.254]	V=46440,<br>p<.001,<br>r=.389
BIC: Thompson+ϵ+η vs Thompson	With ϵ, η: p<.001<br>Without: p=.051	t(579)=-27.682, p<.001,<br>95%CI_m=[-62.395,-54.128], d=1.149,<br>95%CI_d=[1.045,1.255]	V=3089,<br>p<.001,<br>r=.835
ϵ-greedy parameter	SH: p<.001<br>LH: p<.001	t(579)=-11.225, p<.001,<br>95%CI_m=[-0.042,-0.03], d=0.466,<br>95%CI_d=[0.381,0.552]	V=35367,<br>p<.001,<br>r=.503
Novelty bonus η	SH: p<.001<br>LH: p<.001	t(579)=-23.068, p<.001,<br>95%CI_m=[-1.047,-0.883], d=0.958,<br>95%CI_d=[0.86,1.057]	V=10334,<br>p<.001,<br>r=.76
Prior variance σ_0	SH: p<.001<br>LH: p<.001	t(579)=-4.678, p<.001,<br>95%CI_m=[-0.143,-0.058], d=0.194,<br>95%CI_d=[0.112,0.277]	V=54537,<br>p<.001,<br>r=.306
Certain-standard bandit frequency	SH: p<.001<br>LH: p<.001	t(579)=25.974, p<.001,<br>95%CI_m=[6.954,8.091], d=-1.079,<br>95%CI_d=[-1.182,-0.977]	V=157616.5,<br>p<.001,<br>r=.797
Standard bandit frequency	SH: p=.200<br>LH: p<.001	t(579)=15.135, p<.001,<br>95%CI_m=[2.479,3.218], d=-0.628,<br>95%CI_d=[-0.814,-0.54]	V=128795.5,<br>p<.001,<br>r=.543

Supplementary Table 2. Detailed statistics of task effects. p<.05 for the Shapiro normality test indicates that the normality assumption is violated. Student's t-test variables: t=t-statistic, p=p-value, 95%CI_m=95% confidence interval of the difference in the means, d=Cohen's d effect size, 95%CI_d= 95% confidence interval of the effect size. Wilcoxon signed-rank test variables: V=V-statistic, p=p-value, r=effect size. SH: Short horizon condition, LH: Long horizon condition.

		**Certain-standard**		**Standard**		**Novel bandit**	**Low-value bandit**
	**Bandit combination**	**All**	**When is High-value**	**All**	**When is High-value**		
**Initial samples**	[Certain-standard, standard, novel]	5.49 (0.3) [3,10]	5.59 (0.4) [3,10]	5.65 (0.4) [3,10]	6.99 (0.47) [4,10]	-	-
	[Certain-standard, standard, low-value]	5.59 (0.28) [3,10]	5.67 (0.37) [3,10]	5.66 (0.36) [3,10]	7.07 (0.47) [4,10]	-	3.11 (0.26) [2,8]
	[Certain-standard, novel, low-value]	5.5 (0.31) [3,10]		-		-	3.17 (0.29) [2,9]
	[Standard, novel, low-value]	-		5.63 (0.35) [3,10]		-	3.35 (0.28) [2,9]
**Short horizon 1st draw**	[Certain-standard, standard, novel]	6.32 (0.66) [4.5,8.4]	6.34 (0.74) [3.5,8.67]	7.09 (0.81) [3,10]	7.36 (0.74) [5,10]	5.26 (0.67) [3,9]	-
	[Certain-standard, standard, low-value]	5.7 (0.4) [4.69,6.73]	5.66 (0.46) [4.42,6.95]	6.63 (0.59) [4.67,8.38]	6.92 (0.58) [5.09,9]	-	3.13 (0.9) [2,7]
	[Certain-standard, novel, low-value]	6.36 (0.63) [4.86,10]		-		5.32 (0.59) [3.4,7]	3.18 (1.1) [2,10]
	[Standard, novel, low-value]	-		6.99 (0.76) [3,9]		5.32 (0.52) [3,7.67]	3.37 (1.1) [2,10]
**Long horizon 1st draw**	[Certain-standard, standard, novel]	6.55 (0.84) [4,9.2]	6.56 (0.91) [3,9.2]	7.08 (1) [3,10]	7.51 (0.9) [4,10]	5.35 (0.59) [3,9.5]	-
	[Certain-standard, standard, low-value]	5.74 (0.42) [4.74,7.33]	5.7 (0.49) [4.55,7.13]	6.53 (0.59) [4.67,8.11]	6.87 (0.57) [5.14,8.5]	-	3.19 (0.87) [2,8]
	[Certain-standard, novel, low-value]	6.56 (0.73) [3,10]		-		5.42 (0.53) [3,7]	3.28 (1.1) [2,10]
	[Standard, novel, low-value]	-		7.15 (0.95) [3.5,10]		5.46 (0.5) [3,9]	3.34 (0.99) [2,9]
**Long horizon later draws**	[Certain-standard, standard, novel]	6.03 (0.6) [4.18,8.38]	6.01 (0.66) [4,8.5]	6.94 (0.74) [3.67,10]	7.19 (0.69) [3.8,10]	6.23 (0.73) [3,8.73]	-
	[Certain-standard, standard, low-value]	5.69 (0.4) [4.33,7.11]	5.67 (0.46) [3.5,7.33]	6.62 (0.57) [4,8.27]	6.88 (0.57) [4.67,9]	-	3.08 (1) [2,9]
	[Certain-standard, novel, low-value]	6.05 (0.51) [4.52,7.57]		-		6.17 (0.66) [3,10]	3.21 (1.1) [2,9]
	[Standard, novel, low-value]	-		6.53 (0.62) [4.5,8.21]		5.99 (0.58) [3,8.5]	3.16 (1.1) [2,10]

Supplementary Table 3. Additional reward information. Further analysis of the initial samples (1st row; cf. Supplementary Figure 3a-b), further breakdown of the reward associated to the short horizon sample, the long horizon 1st sample, and the average long horizon sample (2nd-4th row; cf. Supplementary Figure 3c). The rewards are broken down according to which 3 out of 4 bandits were shown on these trials, and according to which bandit was the high-value bandit. Data is given as mean (std) [range].

	Model	Thompson				UCB				Hybrid			
			$+\epsilon$	$+\eta$	$+\epsilon$ $+\eta$		$+\epsilon$	$+\eta$	$+\epsilon$ $+\eta$		$+\epsilon$	$+\eta$	$+\epsilon$ $+\eta$
Parameters	Horizon independent	Q_0	Q_0	Q_0	Q_0	Q_0	Q_0	Q_0	Q_0	w, Q_0	w, Q_0	w, Q_0	w, Q_0
	Horizon dependent	σ_0	σ_0, ϵ	σ_0, η	σ_0, ϵ, η	γ, β	γ, β, ϵ	γ, β, η	$\gamma, \beta, \epsilon, \eta$	σ_0, γ, β	$\sigma_0, \gamma, \beta, \epsilon$	$\sigma_0, \gamma, \beta, \eta$	$\sigma_0, \gamma, \beta, \epsilon, \eta$

Supplementary Table 4. Parameters of each model. Table of parameters used for each model compared during model selection (cf. Supplementary Figure 5). Each of the 12 columns indicate a model. The three 'main models' studied were the Thompson model, the UCB model and a hybrid of both. Variants were then created by adding the ϵ-greedy parameter, the novelty bonus and a combination of both. To make sure that the UCB models are not penalized because of the higher number of free parameters compared to the Thompson sampling models, we will also look at the UCB models with fixed inverse temperature parameter (β=1). This leads to a total of 16 models (see the labels on the x-axis in Supplementary Figure 5). All the parameters besides Q_0 and w were fitted to each horizon separately. Parameters: Q_0=prior mean (initial estimate of a bandits mean); σ_0=prior variance (uncertainty about Q_0); w=contribution of UCB vs Thompson; γ =information bonus; β=softmax inverse temperature; ϵ=ϵ-greedy parameter (stochasticity); η=novelty bonus.

	Measure	Analysis	Statistics	BIS		ASRS	
				Bivariate	Partial	Bivariate	Partial
Behaviour	Low-value bandit	Bivariate and partial (correcting for age and IQ) Pearson correlation	Bonferroni corrected for N=3 (p_{cor}) and uncorrected (p_{unc})	r(578)=0.174, p_{cor}<.001, p_{unc}<.001	r(573)=0.118, p_{cor}=.014, p_{unc}=.005	r(578)=0.149, p_{cor}=.001, p_{unc}<.001	r(573)=0.104, p_{cor}=.037, p_{unc}=.012
	Novel bandit			r(578)=0.061, p_{cor}=.431, p_{unc}=.144	r(573)=0.075, p_{cor}=.217, p_{unc}=.072	r(578)=0.086, p_{cor}=.113, p_{unc}=.038	r(573)=0.089, p_{cor}=.101, p_{unc}=.034
	High-value bandit			r(578)=-0.133, p_{cor}=.004, p_{unc}=.001	r(573)=-0.122, p_{cor}=.01, p_{unc}=.003	r(578)=-0.151, p_{cor}=.001, p_{unc}<.001	r(573)=-0.132, p_{cor}=.005, p_{unc}=.002
Model parameters	ϵ-greedy parameter	Bivariate and partial (correcting for age and IQ) Pearson correlation	Bonferroni corrected for N=4 (p_{cor}) and uncorrected (p_{unc})	r(578)=0.171, p_{cor}<.001, p_{unc}<.001	r(573)=0.117, p_{cor}=.019, p_{unc}=.005	r(578)=0.157, p_{cor}=.001, p_{unc}<.001	r(573)=0.115, p_{cor}=.023, p_{unc}=.006
	Novelty bonus η			r(578)=0.061, p_{cor}=.565, p_{unc}=.141	r(573)=0.073, p_{cor}=.32, p_{unc}=.08	r(578)=0.083, p_{cor}=.187, p_{unc}=.047	r(573)=0.087, p_{cor}=.152, p_{unc}=.038
	Prior variance σ_0			r(578)=0.012, p_{cor}=1, p_{unc}=.767	r(573)=0.041, p_{cor}=1, p_{unc}=.331	r(578)=0.015, p_{cor}=1, p_{unc}=.713	r(573)=0.038, p_{cor}=1, p_{unc}=.369
	Prior mean Q_0			r(578)=0.029, p_{cor}=1, p_{unc}=.491	r(573)=0.026, p_{cor}=1, p_{unc}=.531	r(578)=0.03, p_{cor}=1, p_{unc}=.466	r(573)=0.022, p_{cor}=1, p_{unc}=.603

Supplementary Table 5. Detailed correlations between task measures and impulsivity questionnaires. Bivariate and partial (correcting for age and IQ) Pearson's correlation (r), Bonferroni corrected (p_{cor}) and uncorrected p values (p_{unc}). ASRS: Adult ADHD Self-Report Scale, BIS: Barratt Impulsiveness Scale.

	Measure	Analysis	Statistics	Low-value bandit		ϵ-greedy parameter	
				Bivariate	Partial	Bivariate	Partial
BIS subscores	Attentional	Bivariate and partial (correcting for age and IQ) Pearson correlation	Bonferroni corrected for N=3 (p_{cor}) and uncorrected (p_{unc})	r(578)=0.086, p_{cor}=.118, p_{unc}=.039	r(573)=0.054, p_{cor}=.592, p_{unc}=.197	r(573)=0.095, p_{cor}=.067, p_{unc}=.022	r(573)=0.067, p_{cor}=.331, p_{unc}=.110
	Motor			r(578)=0.205, p_{cor}<.001, p_{unc}<.001	r(573)=0.165, p_{cor}<.001, p_{unc}<.001	r(573)=0.198, p_{cor}<.001, p_{unc}<.001	r(573)=0.159, p_{cor}<.001, p_{unc}<.001
	Non-planning			r(578)=0.128, p_{cor}=.006, p_{unc}=.002	r(573)=0.065, p_{cor}=.364, p_{unc}=.121	r(573)=0.120, p_{cor}=.012, p_{unc}=.004	r(573)=0.058, p_{cor}=.501, p_{unc}=.167
ASRS subscores	Hyperactivity-impulsivity	Bivariate and partial (correcting for age and IQ) Pearson correlation	Bonferroni corrected for N=2 (p_{cor}) and uncorrected (p_{unc})	r(578)=0.193, p_{cor}<.001, p_{unc}<.001	r(573)=0.136, p_{cor}=.002, p_{cor}=.001	r(573)=0.205, p_{cor}<.001, p_{unc}<.001	r(573)=0.152, p_{cor}=.001, p_{unc}<.001
	Inattention			r(578)=0.085, p_{cor}=.082, p_{unc}=.041	r(573)=0.057, p_{cor}=.348, p_{unc}=.174	r(573)=0.087, p_{cor}=.074, p_{unc}=.037	r(573)=0.061, p_{cor}=.292, p_{unc}=.146

Supplementary Table 6. Detailed correlations between measures of value-free random exploration and subscales of impulsivity questionnaires. Bivariate and partial Pearson's correlation (r), Bonferroni corrected (p_{cor}) and uncorrected p values (p_{unc}). ASRS: Adult ADHD Self-Report Scale, BIS: Barratt Impulsiveness Scale.

	Measure	Analysis	Statistics	Anxious-depression factor		Uncertainty-related distress factor		Impulsivity factor	
				Bivariate	Partial	Bivariate	Partial	Bivariate	Partial
Behaviour	Low-value bandit	Bivariate and partial (correcting for age and IQ) Pearson correlation	Bon-ferroni corrected for N=9 (p_{cor}) and uncor-rected (p_{unc})	r(578)=-0.058, p_{cor}=1, p_{unc}=.165	r(573)=-0.093, p_{cor}=.232, p_{unc}=.026	r(578)=0.075, p_{cor}=.625, p_{unc}=.069	r(573)=0.035, p_{cor}=1, p_{unc}=.399	r(578)=0.247, p_{cor}<.001, p_{unc}<.001	r(573)=0.191, p_{cor}<.001, p_{unc}<.001
	Novel bandit			r(578)=0.19, p_{cor}<.001, p_{unc}<.001	r(573)=0.17, p_{cor}<.001, p_{unc}<.001	r(578)=0.039, p_{cor}=1, p_{unc}=.343	r(573)=0.037, p_{cor}=1, p_{unc}=.371	r(578)=0.021, p_{cor}=1, p_{unc}=.606	r(573)=0.024, p_{cor}=1, p_{unc}=.567
	High-value bandit			r(578)=-0.174, p_{cor}<.001, p_{unc}<.001	r(573)=-0.138, p_{cor}=.008, p_{unc}=.001	r(578)=-0.09, p_{cor}=.266, p_{unc}=.03	r(573)=-0.073, p_{cor}=0.732, p_{unc}=.081	r(578)=-0.124, p_{cor}=.025, p_{unc}=.003	r(573)=-0.1, p_{cor}=.145, p_{unc}=.016
Model parameters	ϵ-greedy para-meter	Bivariate and partial (correcting for age and IQ) Pearson correlation	Bon-ferroni corrected for N=12 (p_{cor}) and uncor-rected (p_{unc})	r(578)=-0.047, p_{cor}=1, p_{unc}=.262	r(573)=-0.078, p_{cor}=.73, p_{unc}=.061	r(578)=0.107, p_{cor}=.119, p_{unc}=.01	r(573)=0.072, p_{cor}=.997, p_{unc}=.083	r(578)=0.257, p_{cor}<.001, p_{unc}<.001	r(573)=0.204, p_{cor}<.001, p_{unc}<.001
	Novelty bonus η			r(578)=0.14, p_{cor}=.008, p_{unc}<.001	r(573)=0.126, p_{cor}=.03, p_{unc}=.002	r(578)=0.001, p_{cor}=1, p_{unc}=.99	r(573)=-0.002, p_{cor}=1, p_{unc}=.97	r(578)=0.051, p_{cor}=1, p_{unc}=.223	r(573)=0.058, p_{cor}=1, p_{unc}=.167
	Prior variance σ_0			r(578)=0.099, p_{cor}=.203, p_{unc}=.017	r(573)=0.107, p_{cor}=.121, p_{unc}=.01	r(578)=-0.006, p_{cor}=1, p_{unc}=.877	r(573)=0.009, p_{cor}=1, p_{unc}=.821	r(578)=-0.02, p_{cor}=1, p_{unc}=.631	r(573)=0.01, p_{cor}=1, p_{unc}=.816
	Prior mean Q_0			r(578)=0.073, p_{cor}=.925, p_{unc}=.077	r(573)=0.052, p_{cor}=1, p_{unc}=.209	r(578)=0.054, p_{cor}=1, p_{unc}=.197	r(573)=0.048, p_{cor}=1, p_{unc}=.251	r(578)=-0.006, p_{cor}=1, p_{unc}=.891	r(573)=-0.016, p_{cor}=1, p_{unc}=.701

Supplementary Table 7. Detailed correlations between task measures and factor analysis factors. Bivariate and partial Pearson's correlation (r), Bonferroni corrected (p_{cor}) and uncorrected p values (p_{unc}).

Measure	Statistics	Low-value bandit	Novel bandit	High-value bandit
BIS	Bonferroni corrected for N=27 (p_{cor}) and uncorrected (p_{unc})	r(578)=0.174, p_{cor}=.001, p_{unc}<.001	r(578)=0.061, p_{cor}=1, p_{unc}=.144	r(578)=-0.133, p_{cor}=.037, p_{unc}=.001
ASRS		r(578)=0.149, p_{cor}=.008, p_{unc}<.001	r(578)=0.086, p_{cor}=1, p_{unc}=.038	r(578)=-0.151, p_{cor}=.007, p_{unc}<.001
AQ10		r(578)=0.024, p_{cor}=1, p_{unc}=.566	r(578)=0.068, p_{cor}=1, p_{unc}=.103	r(578)=-0.085, p_{cor}=1, p_{unc}=.042
CFS		r(578)=-0.042, p_{cor}=1, p_{unc}=.315	r(578)=-0.133, p_{cor}=.037, p_{unc}=.001	r(578)=0.154, p_{cor}=.005, p_{unc}<.001
OCIR		r(578)=0.18, p_{cor}<.001, p_{unc}<.001	r(578)=-0.05, p_{cor}=1, p_{unc}=0.227	r(578)=-0.033, p_{cor}=1, p_{unc}=.428
STAI		r(578)=0.041, p_{cor}=1, p_{unc}=.319	r(578)=0.145, p_{cor}=.012, p_{unc}<.001	r(578)=-0.17, p_{cor}=.001, p_{unc}<.001
IUS		r(578)=0.078, p_{cor}=1, p_{unc}=.06	r(578)=0.076, p_{cor}=1, p_{unc}=.068	r(578)=-0.127, p_{cor}=.06, p_{unc}=.002
SDS		r(578)=0.092, p_{cor}=.705, p_{unc}=.026	r(578)=0.086, p_{cor}=1, p_{unc}=.038	r(578)=-0.133, p_{cor}=.036, p_{unc}=.001
LSAS		r(578)=-0.076, p_{cor}=1, p_{unc}=.066	r(578)=0.137, p_{cor}=.025, p_{unc}<.001	r(578)=-0.114, p_{cor}=.166, p_{unc}=.006

Supplementary Table 8. Detailed correlations between questionnaires and behavioural task measures. Bivariate Pearson's correlation (r), Bonferroni corrected (p_{cor}) and uncorrected p values (p_{unc}). CFS: Cognitive Flexibility Scale, STAI: State-Trait Anxiety Inventory, IUS: Intolerance of Uncertainty, LSAS: Liebowitz Social Anxiety Scale, SDS: Zung's Self-rating Depression Scale, OCIR: Obsessive-Compulsive Inventory-Revised, AQ10: Autism spectrum Quotient.

Measure	Statistics	ϵ-greedy parameter	Novelty bonus η	Prior variance σ_0	Prior mean Q_0
BIS	Bonferroni corrected for N=36 (p_{cor}) and uncorrected (p_{unc})	r(578)=0.171, p_{cor}=0.001, p_{unc}<.001	r(578)=0.061, p_{cor}=1, p_{unc}=.141	r(578)=0.012, p_{cor}=1, p_{unc}=.767	r(578)=0.029, p_{cor}=1, p_{unc}=.491
ASRS		r(578)=0.157, p_{cor}=.005, p_{unc}<.001	r(578)=0.083, p_{cor}=1, p_{unc}=.047	r(578)=0.015, p_{cor}=1, p_{unc}=.713	r(578)=0.03, p_{cor}=1, p_{unc}=.466
AQ10		r(578)=0.022, p_{cor}=1, p_{unc}=.604	r(578)=0.04, p_{cor}=1, p_{unc}=.34	r(578)=0.042, p_{cor}=1, p_{unc}=.312	r(578)=0.045, p_{cor}=1, p_{unc}=.281
CFS		r(578)=-0.038, p_{cor}=1, p_{unc}=.361	r(578)=-0.095, p_{cor}=.803, p_{unc}=.022	r(578)=-0.052, p_{cor}=1, p_{unc}=.209	r(578)=-0.073, p_{cor}=1, p_{unc}=.078
OCIR		r(578)=0.211, p_{cor}<.001, p_{unc}<.001	r(578)=-0.016, p_{cor}=1, p_{unc}=.705	r(578)=-0.004, p_{cor}=1, p_{unc}=.922	r(578)=-0.019, p_{cor}=1, p_{unc}=.64
STAI		r(578)=0.065, p_{cor}=1, p_{unc}=.116	r(578)=0.118, p_{cor}=.158, p_{unc}=.004	r(578)=0.045, p_{cor}=1, p_{unc}=.283	r(578)=0.041, p_{cor}=1, p_{unc}=.319
IUS		r(578)=0.103, p_{cor}=.466, p_{unc}=.013	r(578)=0.033, p_{cor}=1, p_{unc}=.421	r(578)=0.006, p_{cor}=1, p_{unc}=.881	r(578)=0.063, p_{cor}=1, p_{unc}=.132
SDS		r(578)=0.1, p_{cor}=.559, p_{unc}=.016	r(578)=0.071, p_{cor}=1, p_{unc}=.086	r(578)=0.03, p_{cor}=1, p_{unc}=.465	r(578)=0.036, p_{cor}=1, p_{unc}=.391
LSAS		r(578)=-0.055, p_{cor}=1, p_{unc}=.188	r(578)=0.084, p_{cor}=1, p_{unc}=.044	r(578)=0.119, p_{cor}=.153, p_{unc}=.004	r(578)=0.078, p_{cor}=1, p_{unc}=.06

Supplementary Table 9. Detailed correlations between questionnaires and model parameters. Bivariate Pearson's correlation (r), Bonferroni corrected (p_{cor}) and uncorrected p values (p_{unc}). CFS: Cognitive Flexibility Scale, STAI: State-Trait Anxiety Inventory, IUS: Intolerance of Uncertainty, LSAS: Liebowitz Social Anxiety Scale, SDS: Zung's Self-rating Depression Scale, OCIR: Obsessive-Compulsive Inventory-Revised, AQ10: Autism spectrum Quotient.

BIS	Loadings		
**Items**	**Factor 1**	**Factor 2**	**Factor 3**
1. I plan tasks carefully	0.377	-0.493	0.283
2. I do things without thinking	0.074	-0.214	**0.509**
3. I make-up my mind quickly	-0.304	-0.157	0.135
4. I am happy-go-lucky	-0.456	-0.12	0.18
5. I dont "pay attention"	0.186	-0.081	0.412
6. I have "racing" thoughts	0.06	0.237	0.464
7. I plan trips well ahead of time	0.161	-0.449	0.295
8. I am self controlled	0.423	-0.217	0.328
9. I concentrate easily	**0.501**	-0.123	0.329
10. I save regularly	0.302	-0.247	0.225
11. I "squirm" at plays or lectures	0.076	0.248	0.213
12. I am a careful thinker	0.389	-0.368	0.309
13. I plan for job security	0.487	-0.313	0.108
14. I say things without thinking	-0.007	-0.051	**0.503**
15. I like to think about complex problems	0.306	-0.072	-0.073
16. I change jobs	0.074	-0.005	0.26
17. I act "on impulse"	-0.141	-0.128	**0.618**
18. I get easily bored when solving thought problems	0.115	0.094	0.324
19. I act on the spur of the moment	-0.2	-0.133	**0.599**
20. I am a steady thinker	0.361	-0.243	0.347
21. I change residences	-0.003	0.012	0.169
22. I buy things on impulse	-0.078	-0.004	0.447
23. I can only think about one thing at a time	0.113	0.146	0.07
24. I change hobbies	0.027	0.011	0.308
25. I spend or charge more than I earn	0.074	-0.045	0.485
26. I often have extraneous thoughts when thinking	0.088	0.139	0.456
27. I am more interested in the present than the future	0.001	-0.065	0.15
28. I am restless at the theater or lectures	0.099	0.216	0.306
29. I like puzzles	0.195	-0.077	-0.009
30. I am future oriented	0.471	-0.275	0.005

Supplementary Table 10. Items from the BIS and their loading score on each factor extracted from the factor analysis. Factor 1: Anxious-Depression, Factor 2: Uncertainty-related distress, Factor 3: Impulsivity. Larger weights (arbitrary value >0.5) are indicated.

ASRS	Loadings		
**Items**	**Factor 1**	**Factor 2**	**Factor 3**
1. How often do you have trouble wrapping up the final details of a project, once the challenging parts have been done?	0.181	0.095	0.416
2. How often do you have difficulty getting things in order when you have to do a task that requires organization?	0.261	0.018	0.446
3. How often do you have problems remembering appointments or obligations?	0.143	-0.017	0.437
4. When you have a task that requires a lot of thought, how often do you avoid or delay getting started?	0.395	0.036	0.261
5. How often do you fidget or squirm with your hands or feet when you have to sit down for a long time?	0.081	0.125	0.436
6. How often do you feel overly active and compelled to do things, like you were driven by a motor?	-0.23	0.238	0.484
7. How often do you make careless mistakes when you have to work on a boring or difficult project?	0.183	-0.022	0.468
8. How often do you have difficulty keeping your attention when you are doing boring or repetitive work?	0.185	0.021	0.476
9. How often do you have difficulty concentrating on what people say to you, even when they are speaking to you directly?	0.2	0.13	0.413
10. How often do you misplace or have difficulty finding things at home or at work?	0.067	0.022	0.477
11. How often are you distracted by activity or noise around you?	0.173	0.097	0.47
12. How often do you leave your seat in meetings or other situations in which you are expected to remain seated?	-0.039	0.094	**0.533**
13. How often do you feel restless or fidgety?	0.073	0.19	0.497
14. How often do you have difficulty unwinding and relaxing when you have time to yourself?	0.099	0.335	0.349
15. How often do you find yourself talking too much when you are in social situations?	-0.089	0.086	0.47
16. When you're in a conversation, how often do you find yourself finishing the sentences of the people you are talking to, before they can finish them themselves?	-0.128	0.176	0.398
17. How often do you have difficulty waiting your turn in situations when turn taking is required?	-0.069	0.181	**0.506**
18. How often do you interrupt others when they are busy?	-0.08	0.112	0.461

Supplementary Table 11. Items from the ASRS and their loading score on each factor extracted from the factor analysis. Factor 1: Anxious-Depression, Factor 2: Uncertainty-related distress, Factor 3: Impulsivity. Larger weights (arbitrary value >0.5) are indicated.

AQ10	Loadings		
**Items**	**Factor 1**	**Factor 2**	**Factor 3**
1. I often notice small sounds when others do not	0.03	-0.152	-0.189
2. I usually concentrate more on the whole picture, rather than the small details	0.244	0.035	-0.044
3. I find it easy to do more than one thing at once	0.345	0.022	-0.026
4. If there is an interruption, I can switch back to what I was doing very quickly	0.422	0.073	0.154
5. I find it easy to "read between the lines" when someone is talking to me	0.208	0.03	0.093
6. I know how to tell if someone listening to me is getting bored	0.065	0.134	0.079
7. When I'm reading a story, I find it difficult to work out the characters intentions	-0.075	-0.109	-0.155
8. I like to collect information about categories of things (e.g., types of car, types of bird, types of train, types of plant etc)	0.093	-0.201	-0.179
9. I find it easy to work out what someone is thinking or feeling just by looking at their face	0.207	-0.035	0.008
10. I find it difficult to work out people's intentions	-0.243	-0.075	-0.095

Supplementary Table 12. Items from the AQ10 and their loading score on each factor extracted from the factor analysis. Factor 1: Anxious-Depression, Factor 2: Uncertainty-related distress, Factor 3: Impulsivity. Larger weights (arbitrary value >0.5) are indicated.

CFS	Loadings		
Items	Factor 1	Factor 2	Factor 3
1. I can communicate an idea in many different ways	**-0.512**	-0.013	0.079
2. I avoid new and unusual situations	-0.441	-0.391	0.114
3. I feel like I never get to make decisions	-0.305	-0.246	-0.195
4. I can find workable solutions to seemingly unsolvable problems	-0.446	0.126	0.119
5. I seldom have choices when deciding how to behave	-0.087	-0.241	-0.194
6. I am willing to work at creative solutions to problems	-0.325	-0.001	0.099
7. In any given situation, I am able to act appropriately	-0.38	0.053	-0.218
8. My behaviour is a result of conscious decisions that I make	-0.304	0.103	-0.231
9. I have many possible ways of behaving in any given situation	-0.257	-0.03	0.093
10. I have difficulty using my knowledge on a given topic in real life situations	-0.396	-0.179	-0.081
11. I am willing to listen and consider alternatives for handling a problem	-0.262	0.068	-0.165
12. I have the self-confidence necessary to try different ways of behaving	**-0.68**	-0.014	0.098

Supplementary Table 13. Items from the CFS and their loading score on each factor extracted from the factor analysis. Factor 1: Anxious-Depression, Factor 2: Uncertainty-related distress, Factor 3: Impulsivity. Larger weights (arbitrary value >0.5) are indicated.

OCIR	Loadings		
Items	Factor 1	Factor 2	Factor 3
1. I have saved up so many things that they get in the way	0.147	0.165	0.383
2. I check things more often than necessary	0.001	0.347	0.409
3. I get upset if objects are not arranged properly	-0.157	0.47	0.304
4. I feel compelled to count while I am doing things	-0.113	0.348	0.31
5. I find it difficult to touch an object when I know it has been touched by strangers or certain people	-0.059	0.298	0.201
6. I find it difficult to control my own thoughts	0.207	0.248	**0.517**
7. I collect things I don't need	0.109	0.124	0.419
8. I repeatedly check doors, windows, drawers, etc	-0.148	0.36	0.287
9. I get upset if others change the way I have arranged things	-0.032	0.455	0.254
10. I feel I have to repeat certain numbers	-0.071	0.244	0.314
11. I sometimes have to wash or clean myself simply because I feel contaminated	-0.084	0.277	0.283
12. I am upset by unpleasant thoughts that come into my mind against my will	0.153	0.222	**0.508**
13. I avoid throwing things away because I am afraid I might need them later	0.053	0.226	0.357
14. I repeatedly check gas and water taps and light switches after turning them off	-0.167	0.397	0.267
15. I need things to be arranged in a particular way	-0.188	**0.53**	0.238
16. I feel that there are good and bad numbers	-0.046	0.28	0.255
17. I wash my hands more often and longer than necessary	-0.191	0.305	0.319
18. I frequently get nasty thoughts and have difficulty in getting rid of them	0.197	0.179	**0.53**

Supplementary Table 14. Items from the OCIR and their loading score on each factor extracted from the factor analysis. Factor 1: Anxious-Depression, Factor 2: Uncertainty-related distress, Factor 3: Impulsivity. Larger weights (arbitrary value >0.5) are indicated.

SDS	Loadings		
**Items**	**Factor 1**	**Factor 2**	**Factor 3**
1. I feel down-hearted and blue	0.496	0.116	0.263
2. Morning is when I feel the best (-)	0.283	-0.119	0.007
3. I have crying spells or feel like it	0.275	0.167	0.263
4. I have trouble sleeping at night	0.173	0.13	0.276
5. I eat as much as I used to (-)	0.077	0.046	0.074
6. I still enjoy sex (-)	0.381	0.058	-0.015
7. I notice that I am losing weight	-0.15	0.098	0.155
8. I have trouble with constipation	0.032	0.092	0.253
9. My heart beats faster than usual	0.115	0.23	0.268
10. I get tired for no reason	0.348	0.14	0.29
11. My mind is as clear as it used to be (-)	**0.563**	-0.018	0.191
12. I find it easy to do the things I used to (-)	**0.587**	-0.053	0.181
13. I am restless and can't keep still	-0.041	0.22	**0.5**
14. I feel hopeful about the future (-)	**0.656**	0.015	-0.043
15. I am more irritable than usual	0.188	0.233	0.327
16. I find it easy to make decisions (-)	**0.571**	0.086	0.093
17. I feel that I am useful and needed (-)	**0.711**	-0.004	0.062
18. My life is pretty full (-)	**0.619**	0.04	-0.073
19. I feel that others would be better off if I were dead	0.247	0.102	0.247
20. I still enjoy the things I used to do (-)	**0.572**	0.07	0.133

Supplementary Table 15. Items from the SDS and their loading score on each factor extracted from the factor analysis. Factor 1: Anxious-Depression, Factor 2: Uncertainty-related distress, Factor 3: Impulsivity. Larger weights (arbitrary value >0.5) are indicated.

STAI	Loadings		
**Items**	**Factor 1**	**Factor 2**	**Factor 3**
1. I feel pleasant (-)	**0.678**	-0.03	0.066
2. I feel nervous and restless	0.436	0.181	0.295
3. I feel satisfied with myself (-)	**0.724**	-0.03	0.045
4. I wish I could be as happy as others seem to be	0.461	0.162	0.185
5. I feel like a failure	**0.608**	0.08	0.181
6. I feel rested (-)	0.448	0.071	0.169
7. I am calm, cool, and collected (-)	**0.554**	0.017	0.195
8. I feel that difficulties are piling up so that I cannot overcome them	0.411	0.18	0.306
9. I worry too much over something that really doesnt matter	0.278	0.272	0.263
10. I am happy (-)	**0.691**	-0.006	0.065
11. I have disturbing thoughts	0.232	0.132	0.449
12. I lack self confidence	**0.635**	0.135	0
13. I feel secure (-)	**0.632**	-0.001	0.146
14. I make decision easily (-)	**0.553**	0.024	0.074
15. I feel inadequate	**0.626**	0.033	0.14
16. I am content (-)	**0.672**	-0.034	0.105
17. Some unimportant thoughts runs through my mind and bothers me	0.201	0.232	0.429
18. I take disappointments so keenly that I can't put them out of my mind	0.316	0.292	0.265
19. I am a steady person (-)	**0.577**	-0.084	0.276
20. I get in a state of tension or turmoil as I think over my recent concerns and interests	0.287	0.248	0.386

Supplementary Table 16. Items from the STAI and their loading score on each factor extracted from the factor analysis. Factor 1: Anxious-Depression, Factor 2: Uncertainty-related distress, Factor 3: Impulsivity. Larger weights (arbitrary value >0.5) are indicated.

IUS	Loadings		
**Items**	**Factor 1**	**Factor 2**	**Factor 3**
1. Uncertainty stops me from having a firm opinion	0.289	0.305	0.164
2. Being uncertain means that a person is disorganized	-0.059	0.347	0.113
3. Uncertainty makes life intolerable	0.004	**0.547**	0.113
4. It's unfair not having any guarantees in life	0.075	0.447	0.165
5. My mind can't be relaxed if I dont know what will happen tomorrow	0.001	**0.706**	0.072
6. Uncertainty makes me uneasy, anxious, or stressed	0.183	**0.688**	0.052
7. Unforeseen events upset me greatly	0.092	**0.755**	0.06
8. It frustrates me not having all the information I need	0.046	**0.639**	0.017
9. Uncertainty keeps me from living a full life	0.213	**0.645**	0.034
10. One should always look ahead so as to avoid surprises	-0.102	**0.591**	-0.199
11. A small unforeseen event can spoil everything, even with the best of planning	0.07	**0.505**	-0.043
12. When it's time to act, uncertainty paralyses me	0.209	**0.573**	0.122
13. Being uncertain means that I am not first rate	0.108	**0.601**	0.093
14. When I am uncertain, I can't go forward	0.175	**0.636**	0.046
15. When I am uncertain I can't function very well	0.18	**0.642**	0.111
16. Unlike me, others always seem to know where they are going with their lives	0.405	0.375	0.038
17. Uncertainty makes me vulnerable, unhappy, or sad	0.238	**0.662**	0.082
18. I always want to know what the future has in store for me	-0.055	**0.661**	-0.014
19. I can't stand being taken by surprise	0.132	**0.628**	-0.092
20. The smallest doubt can stop me from acting	0.264	**0.573**	0.051
21. I should be able to organize everything in advance	-0.126	**0.754**	-0.117
22. Being uncertain means that I lack confidence	0.303	**0.512**	-0.001
23. I think it's unfair that other people seem sure about their future	0.187	0.485	0.097
24. Uncertainty keeps me from sleeping soundly	0.116	**0.537**	0.22
25. I must get away from all uncertain situations	0.03	**0.721**	0.04
26. The ambiguities in life stress me	0.22	**0.666**	0.095
27. I can't stand being undecided about my future	0.113	**0.664**	0.102

Supplementary Table 17. Items from the IUS and their loading score on each factor extracted from the factor analysis. Factor 1: Anxious-Depression, Factor 2: Uncertainty-related distress, Factor 3: Impulsivity. Larger weights (arbitrary value >0.5) are indicated.

LSAS	Loadings		
**Items**	**Factor 1**	**Factor 2**	**Factor 3**
1. Telephone in public	0.474	0.219	-0.076
2. Participating in small groups	**0.538**	0.225	-0.082
3. Eating in public place	0.383	0.144	-0.068
4. Drinking with others in public places	0.352	0.196	-0.074
5. Talking to people in authority	0.483	0.181	0.002
6. Acting, performing or giving a talk in front of an audience	**0.571**	0.17	-0.232
7. Going to a party	**0.538**	0.222	-0.22
8. Working while being observed	0.418	0.165	0.024
9. Writing while being observed	0.287	0.146	0.087
10. Calling someone you don’t know very well	**0.577**	0.134	-0.017
11. Talking with people you don’t know very well	**0.595**	0.194	-0.127
12. Meeting strangers	**0.564**	0.236	-0.179
13. Urinating in a public bathroom	0.177	0.112	-0.081
14. Entering a room when others are already seated	0.403	0.223	-0.077
15. Being the centre of attention	**0.577**	0.213	-0.257
16. Speaking up at a meeting	**0.582**	0.176	-0.161
17. Taking a test	0.323	0.205	0.029
18. Expressing a disagreement or disapproval to people you don’t know very well	0.47	0.176	-0.044
19. Looking at people you don’t very well in the eyes	0.443	0.153	0.05
20. Giving a report to a group	**0.557**	0.174	-0.109
21. Trying to pick up someone	0.477	0.055	-0.132
22. Returning goods to a store	0.464	0.09	-0.021
23. Giving a party	**0.532**	0.136	-0.143
24. Resisting a high pressure salesperson	0.3	0.106	0.051

Supplementary Table 18. Items from the LSAS and their loading score on each factor extracted from the factor analysis. Factor 1: Anxious-Depression, Factor 2: Uncertainty-related distress, Factor 3: Impulsivity. Larger weights (arbitrary value >0.5) are indicated.

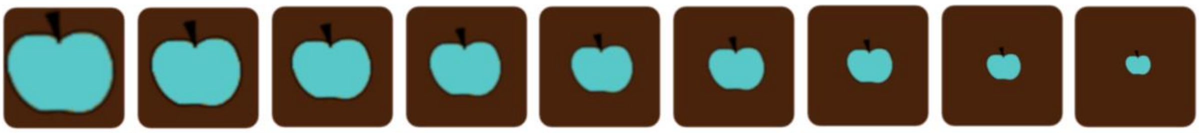

Supplementary Figure 1. Visualisation of the 9 different sizes that the apples can take. The associated rewards go from 2 (small apple on the right) to 10 (big apple on the left). The reward is given by linearly increasing the radius of the apple.

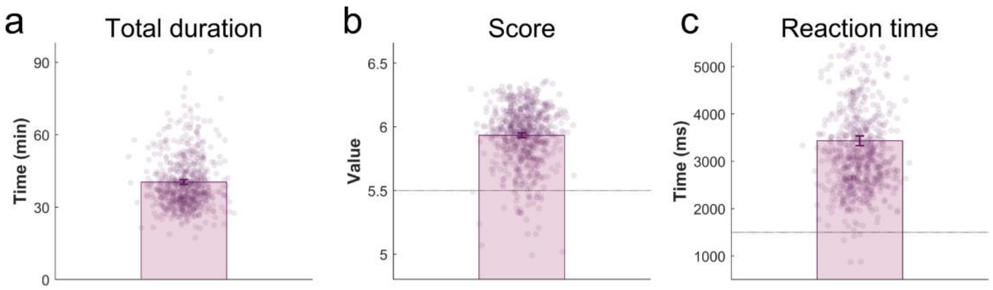

Supplementary Figure 2. Exclusion criteria applied on total data (N=657). (a) Total task duration. Participants are excluded if (b) their total score (average apple size) was lower than 5.5 (horizontal line), if (c) their reaction time on the 1st choice was lower than 1500ms (horizontal line) or if they failed at least one attention check in the questionnaires. According to those exclusion criteria, N=77 participants were excluded from further analyses, resulting in a final sample of N=580 participants. Data is shown as mean ± 95%CI and each dot represent one participant. Source data are provided as a Source Data file. Bar values: Total duration: 40.396; Score: 5.933; Reaction time: 3.434e+03.

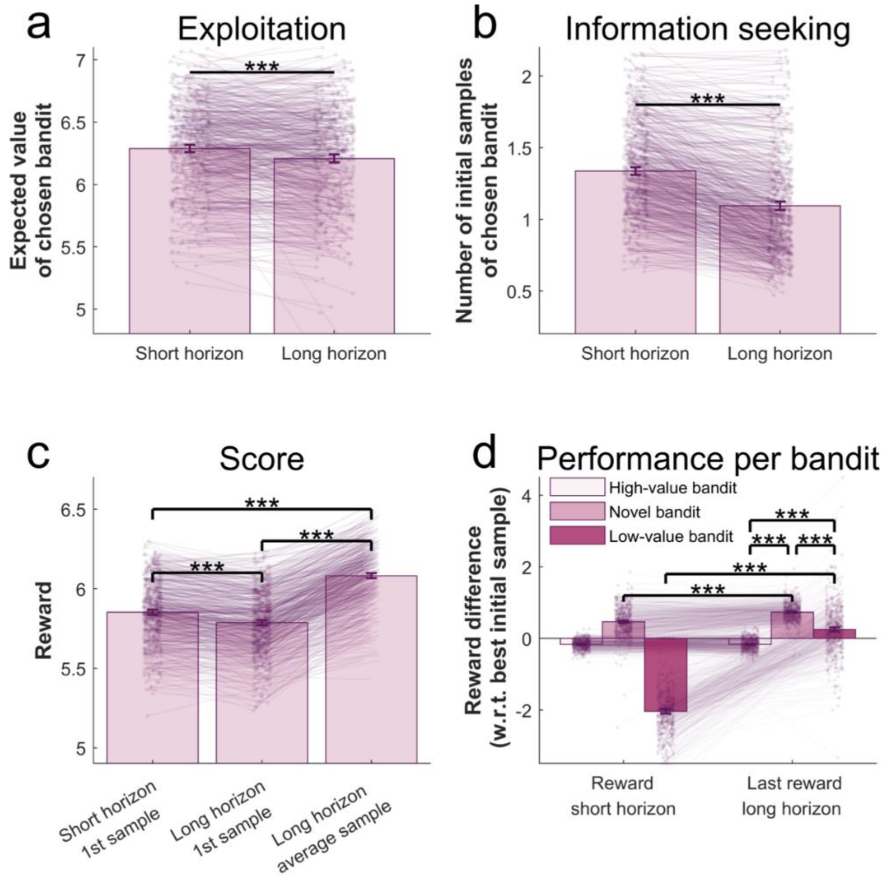

Supplementary Figure 3. Benefits of exploration (N=580). Effect of information on performance. (a) The first bandit participants chose as a function of its expected value (average of its initial samples). Participants chose bandits with a lower expected value (i.e., they exploited less) in the long horizon compared to the short horizon (two-sided Wilcoxon signed-rank test: V=110057, p=1.627e-10, Wilcoxon effect size r=0.265). (b) The first bandit participants chose as a function of the number of samples that were initially revealed. Participants chose less known (i.e., more informative) bandits in the long compared to the short horizon (V=160109.5, p=9.087e-82, r=0.796). (c) The first draw in the long horizon led to a lower reward than the first draw in the short horizon (V=131612, p=2.306e-37, r=0.53), indicating that participants sacrificed larger initial outcomes for the benefit of more information. This additional information helped making better decisions in the long run, leading to a higher earning over all draws in the long horizon (V=264, p=4.315e-96, r=0.864). (d) Splitting the data by participant's first choice, either high-value bandit (exploitation), novel bandit (exploration) or low-value bandit (exploration) revealed that the improved performance (when looking at the long horizon's final choice relative to the best initial sample) following exploration is not strategy-specific (paired-samples t-test: reward short horizon vs last reward long horizon: High-value bandit: t(512)=0.246, p=0.806, d=0.011, novel bandit: t(512)=-15.8, p=4.281e-46, d=0.698, low-value bandit: t(488)=-49.213, p=2.419e-191, d=2.226; Last reward long horizon: High-value bandit vs novel bandit: t(512)=-69.411, p=1.262e-262, d=3.065, high-value bandit vs low-value bandit: t(501)=-13.513, p=1.062e-35, d=0.60, novel bandit vs low-value bandit: t(501)=14.786, p=2.589e-41, d=0.66). For the effect of trial on score cf. Supplementary Figure 4. For detailed statistics cf. Supplementary Table 2. *** *p*<.001. Data are shown as mean ± 95%CI and each dot/line represent one participant. Sample size for statistics: N=580 human participants. Source data are provided as a Source Data file. Bar values: Exploitation: Short Horizon (SH): 6.288, Long Horizon (LH): 6.209; Information Seeking: SH: 1.337; LH: 1.095; Score: SH: 5.853, LH 1st sample: 5.787, LH average sample: 6.082; Performance difference: SH: High-value bandit: -0.163, Novel bandit: 0.468, Low-value bandit: -2.034; LH: High-value bandit: -0.165, Novel bandit: 0.734, Low-value bandit: 0.248.

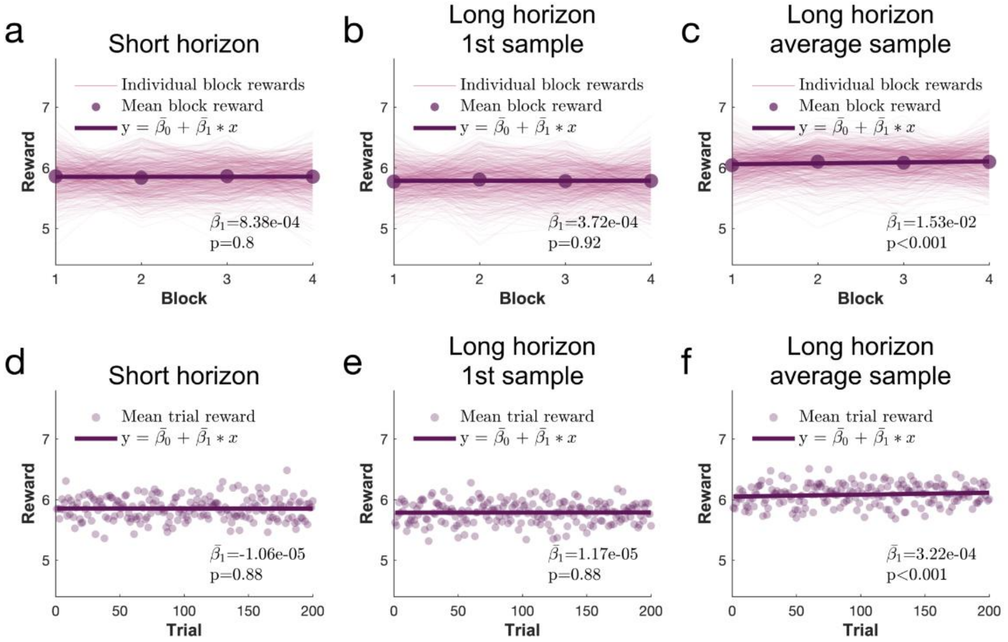

Supplementary Figure 4. Influence of block and trial on score. For each participant, we performed a regression on the average reward per block as well as a regression on the reward per trial. Slopes were compared to a vector of null slopes. When analysing the reward per block: (a) No slope was observed for the short horizon reward (slope=8.38e-04, one-sample t-test: t(579)=0.249, p=0.804, d=0.01, 95%CI=[-0.0058,0.0075]) not in (b) the long horizons' first sample reward (slope=3.72e-04, t(579)=0.104, p=0.917, d=0.004, 95%CI=[-0.0067,0.0074]). A mild increase in (c) the long horizon's average reward (slope=1.53e-02, t(579)=5.409, p=9.266e-08, d=0.225, 95%CI=[0.0098,0.0209]) was observed. Similarly, when analysing the reward per trial: (d) No slope was observed for the short horizon reward (slope=-1.06e-05, t(579)=-0.153, p=0.878, 95%CI=[-1.47e-04,1.26e-04]) nor in (e) the long horizons' first sample reward (slope= 1.17e-05, t(579)=0.156, p=0.876, 95%CI=[-1.36e-04,1.59e-04]). A mild increase in (f) the long horizon's average reward (slope=3.22e-04, t(579)=5.24, p=2.237e-07, 95%CI=[2.02e-04,4.43e-04]) was observed. Source data are provided as a Source Data file.

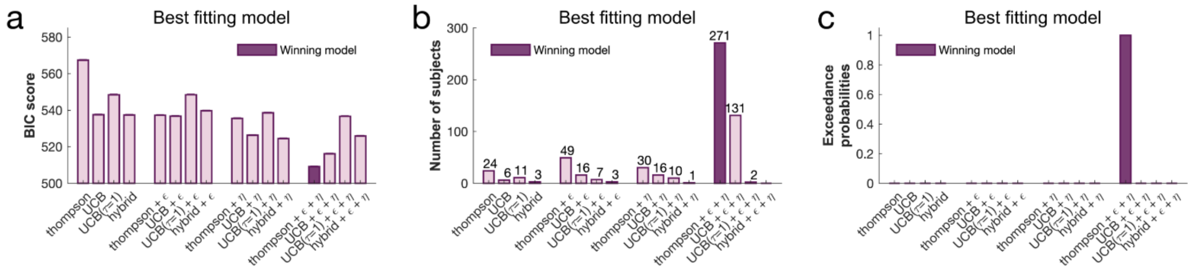

Supplementary Figure 5. Model comparison (N=580). Participants use a mixture of exploration strategies. 16 models were compared. Bayesian Information Criterion (BIC) was used for model selection (cf. Supplementary Table 2 for statistics). The Thompson model with both the ϵ-greedy parameter and the novelty bonus η performed best when comparing (a) average BIC score, (b) the number of participants best fitted by each model and (c) the exceedance probabilities when using Bayesian model selection. Source data are provided as a Source Data file. Bar values: BIC scores: from left to right: 567.367, 537.525, 548.405, 537.397, 537.248, 536.680, 548.466, 539.738, 535.511, 526.277, 538.578, 524.445, 509.106, 516.159, 536.649, 525.857.

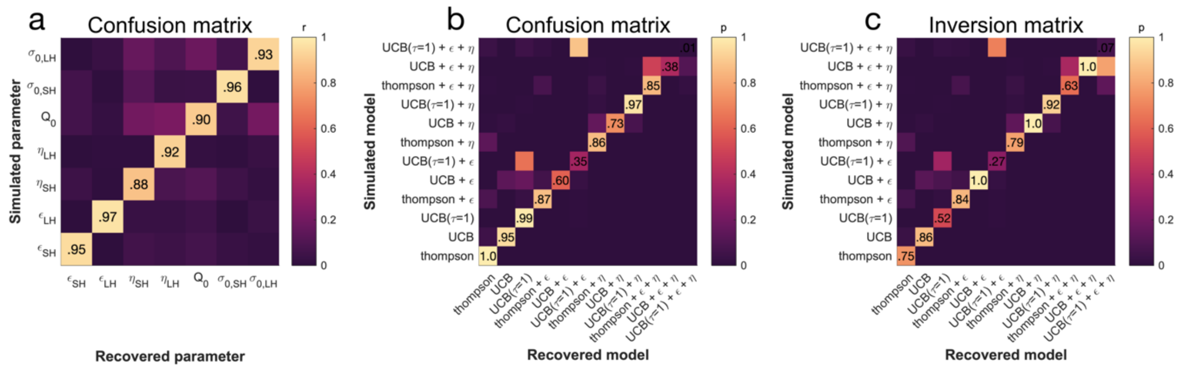

Supplementary Figure 6. Confusion matrices. (a) Confusion matrix of Parameter recovery. For each parameter of the winning model (Thompson+ η +ϵ from the pilot data), we sampled parameter values from a normal distribution defined by the pilot data mean and standard deviation, which we used to simulate behaviour (for additional simulations cf. Supplementary Figure 19 and Supplementary Figure 24). This was performed N=20000 times. For each simulation, we fitted the model and computed the Pearson correlation *r* between the simulated parameters and the fitted parameters. (b) Confusion matrix of model identification: p(fit model|simulated model). For each model, behaviour was simulated N=100 times with parameter values sampled from the pilot data mean and standard deviation. All models were fitted to this simulated data and BIC scores compared. The percentage *p* of how often each fitted model won given the simulated model was computed. The lower recovery of the full-UCB model is likely to reflects the conservative nature of the BIC which punishes its high complexity to the advantage of the simpler (but equally versatile) full-Thompson model. (c) Inversion matrix of model identification: p(simulated model|fit model). The percentage *p* of how often the data was simulated by a specific model given the fitted model was computed. Source data are provided as a Source Data file.

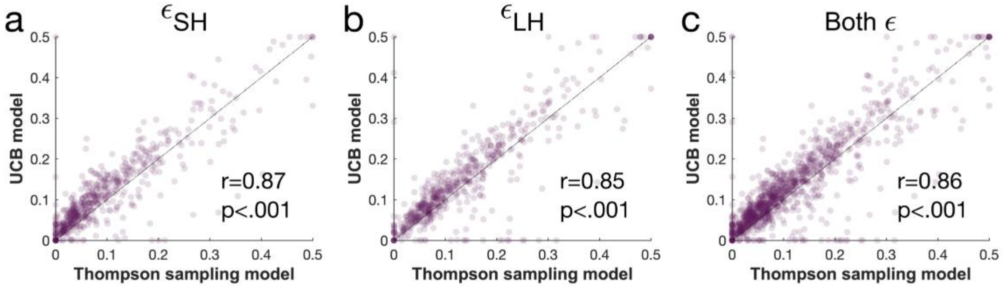

Supplementary Figure 7. Epsilon correlations across complex models. Pearson correlation between ϵ values from the Thompson+ϵ+η model and the UCB+ϵ+η model. (a) Short horizon ϵ values. (b) Long horizon ϵ values. (c) Both horizon conditions concatenated. All three p-values were approximated to zero on Matlab R2021b indicating that they are too small to be computed (below the lower bound of the corr function). Source data are provided as a Source Data file.

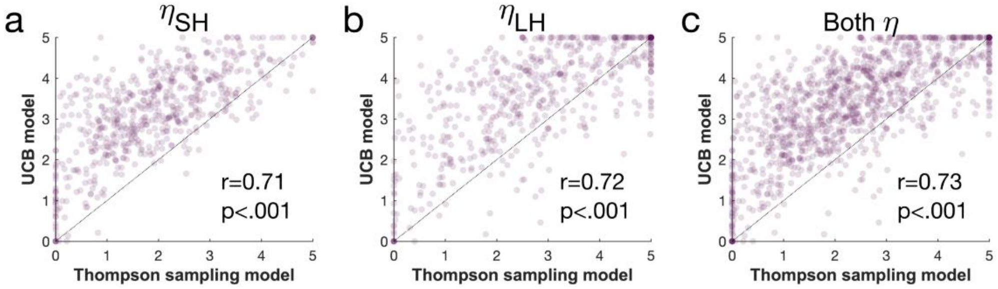

Supplementary Figure 8. Eta correlations. Pearson correlation between η values from the Thompson+ϵ+η model and the UCB+ϵ+η model. (a) Short horizon η. (b) Long horizon η values. (c) Both horizon conditions concatenated. All three p-values were approximated to zero on Matlab R2021b indicating that they are too small to be computed (below the lower bound of the corr function). Source data are provided as a Source Data file.

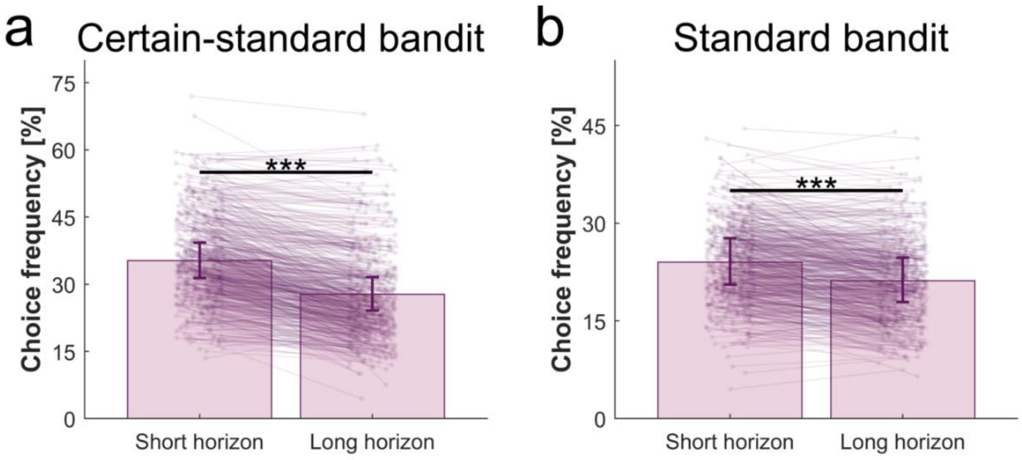

Supplementary Figure 9. Further behavioural horizon effects (N=580). Choice patterns in the first draw for each horizon condition. Participants sampled from the (a) certain-standard bandit (i.e., bandit with 3 initial samples) more in the short horizon compared to the long horizon (two-sided Wilcoxon signed-rank test: V=153594.5, p=2.181e-83, Wilcoxon effect size r=0.809) and from the (b) standard bandit (i.e., bandit with 1 initial sample) more in the short horizon compared to the long horizon (V=134660, p=2.413e-52, r=0.633). *** p<.001. For detailed statistics cf. Supplementary Table 2. Data are shown as mean ± binomial 95%CI and each dot/line represent one participant. Source data are provided as a Source Data file. Bar values: Certain-standard bandit: Short Horizon (SH): 35.272, Long Horizon (LH): 27.749; Standard bandit: SH: 24.000, LH: 21.152.

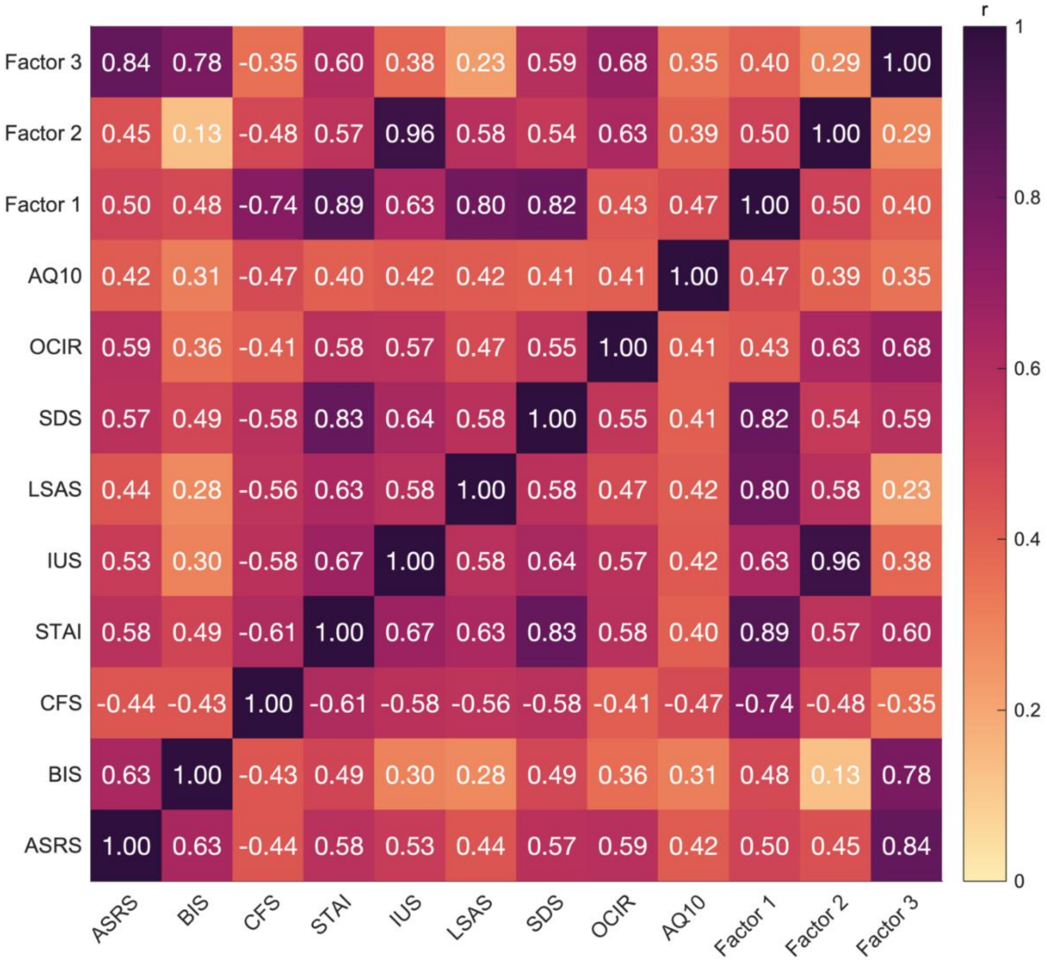

Supplementary Figure 10. Questionnaire and factor correlations. Pearson correlations between participants’ (N=580) questionnaire scores. ASRS: Adult ADHD Self-Report Scale, BIS: Barratt Impulsiveness Scale, CFS: Cognitive Flexibility Scale, STAI: State-Trait Anxiety Inventory, IUS: Intolerance of Uncertainty, LSAS: Liebowitz Social Anxiety Scale, SDS: Zung's Self-rating Depression Scale, OCIR: Obsessive-Compulsive Inventory-Revised, AQ10: Autism spectrum Quotient, Factor 1: Anxious-Depression factor, Factor 2: Uncertainty-related distress, Factor 3: Impulsivity factor. Source data are provided as a Source Data file.

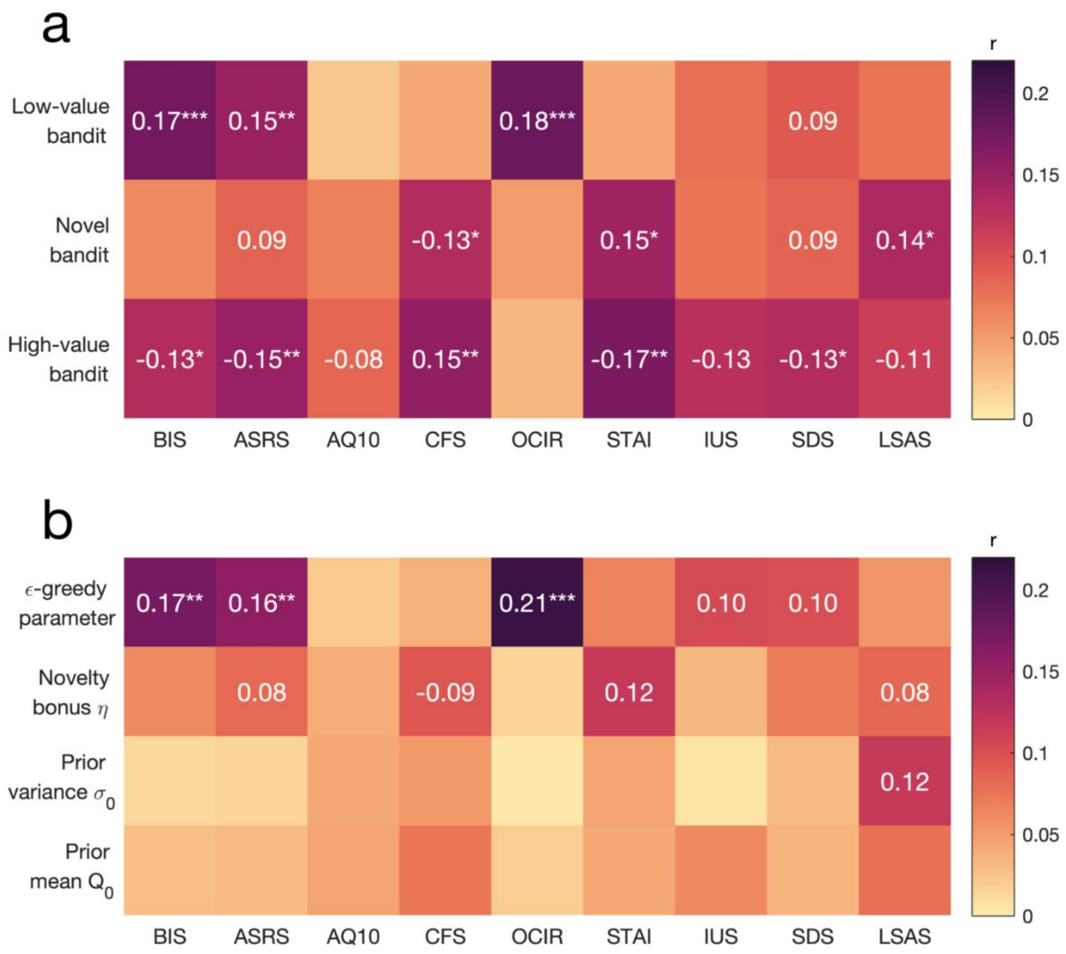

Supplementary Figure 11. Questionnaire and task measure correlations. Pearson correlations between participants' (N=580) questionnaire scores and (a) behavioral task measures (i.e. bandit picking frequencies) and (b) model parameters. Significant (uncorrected) correlations are displayed. The asterisks indicate the significance of the Bonferroni corrected (for (a) N=27 and (b) N=36) correlations. ASRS: Adult ADHD Self-Report Scale, BIS: Barratt Impulsiveness Scale, CFS: Cognitive Flexibility Scale, STAI: State-Trait Anxiety Inventory, IUS: Intolerance of Uncertainty, LSAS: Liebowitz Social Anxiety Scale, SDS: Zung's Self-rating Depression Scale, OCIR: Obsessive-Compulsive Inventory-Revised, AQ10: Autism spectrum Quotient. *=p_{cor}<.05, **=p_{cor}<.01, ***=p_{cor}<.001. Source data are provided as a Source Data file.

Supplementary Note 2: Pilot experiment

Methods and results

Participant recruitment

To take part in the study, participants had to be above 18 years of age and have their current residence in the UK. To ensure data quality, participants were be excluded according to the following criteria: data is incomplete, the mean score (i.e., apple size) is lower than 5.5 indicating participants were performing at chance level (cf. Supplementary Figure 13b), the first draw mean reaction time was faster than 1500ms indicating participants are not allocating much thought to their choice (cf. Supplementary Figure 13c) and if participants failed at least one attention check during the questionnaires meaning that they were not reading the questions. According to these exclusion criteria, N=3 participants were excluded from the pilot data (cf. Supplementary Figure 13).

Participants explore more when it is worth it

Participants chose bandits with a lower expected value (computed as the mean of the bandits' initial samples) in the long horizon (pilot data: t(60)=3.585, p=.001, 95% confidence interval of the mean: CI_M=[0.047,0.165], effect size: Cohen's d=-0.459, 95% confidence interval of the effect size CI_ES=[-0.727,-0.195]; Supplementary Figure 14a). This was reflected on the frequency of picking the high-value bandit, which was decreased in the long horizon (pilot data: t(60)=8.45, p<.001, 95%CI_M=[6.92,11.211], d=-1.082, 95%CI_ES=[-1.407,-0.769]; Supplementary Figure 16a), and similarly on the frequency of picking the low-value bandit, which was increased in the long horizon (pilot data: t(60)=-3.446, p=.001, 95%CI_M=[-1.568,-0.416], d=0.441, 95%CI_ES=[0.178,0.708]; Supplementary Figure 16c). This exploration was goal-directed, with participants choosing bandits they know less about (lower number of initial samples, i.e. more informative) in the long horizon (pilot data: t(60)=9.625, p<.001, 95%CI_M=[0.184,0.281], d=-1.232, 95%CI_ES=[-1.576,-0.903]; Supplementary Figure 14b). This was largely reflected on the frequency of the novel bandit, which was increased in the long horizon (pilot data t(60)=-8.586, p<.001, 95%CI_M=[-11.178,-6.954], d=1.099, 95%CI_ES=[0.784,1.427]; Supplementary Figure 16c).

Participants use exploration beneficially

To evaluate whether participants were able to use exploration beneficially, we looked at their performance (i.e. the outcomes they obtained). We first compared the reward (i.e. apple size) obtained in the short horizon with the first reward obtained in the long horizon. The latter was lower (pilot data: t(60)=6.522, p<.001, 95%CI_M=[0.059,0.112], d=-0.835, 95%CI_ES=[-1.134,-0.545]; Supplementary Figure 14c, Supplementary Table 20) which is a reflection of exploration. The long horizon average reward (across 6 draws) was higher than the short horizon reward (pilot data: t(60)=-16.096, p<.001, 95%CI_M=[-0.245,-0.191], d=2.061, 95%CI_ES=[1.626,2.524]; Supplementary Figure 14c), indicating that participants made good use of the additional information earned by exploring.

Participants explore using heuristics

To formally assess which exploration strategies are being used, we turned to computational modeling. Similar to the behavioural analysis, only the first draw of each trial was analysed. We compared 16 models that make different predictions about the usage of exploration strategies (cf. Supplementary Information, Model Descriptions). Participants used a mixture of computationally demanding (i.e., Thompson sampling and/or UCB) and heuristic exploration strategies (i.e., value-free random exploration and novelty exploration) captured by the winning model (pilot data: BIC

average score: Thompson+η+ϵ vs Thompson model: t(60)=-10.187, p<.001, 95%CI_M=[-72.866,-48.946], d=1.304, 95%CI_ES=[0.967,1.657]; Supplementary Figure 19, Supplementary Table 20). Model comparison was computed using the commonly used Bayesian Information Criterion (BIC). The winning model, i.e. the model with the lowest BIC score, was used for subsequent analyses.

Participants rely more on heuristics in the long horizon

To assess the changes in exploration strategy, we examined the winning model's fitted parameters. Those parameters were fitted to the first draw of all trials of each participant. The ϵ-greedy parameter, which captures the contribution of value-free random exploration, was increased in the long (versus short) horizon (pilot data: t(60)=-3.23, p=.002, 95%CI_M=[-0.058,-0.014], d=0.413, 95%CI_ES=[0.152,0.679]; Supplementary Figure 21a, Supplementary Table 20). The novelty bonus η, which captures the intrinsic reward of selecting a novel option, was increased in the long horizon (pilot data: t(60)=-9.43, p<.001, 95%CI_M=[-1.265,-0.822], d=1.207, 95%CI_ES=[0.881,1.548]; Supplementary Figure 21b).

Further analysis of the high-value bandit frequency

The horizon effect on the frequency of picking the high-value bandit was independent of whether the high-value bandit had 3 prior samples (pilot data: certain-standard bandit: t(61)=-9.092, p<.001 d=1.164, 95%CI=[-7.23,-4.622]) or 1 prior sample (standard bandit: pilot data: t(61)=-5.825, p<.001, d=.746, 95%CI=[-4.217,-2.061]) associated to it (samples main effect: F(1, 60)=83.248, p<.001, η^2=.581). Interestingly this horizon effect was stronger in the former (samples-by-horizon interaction effect: F(1, 60)=27.767, p<.001, η^2=.316), in line with predictions from uncertainty-guided exploration strategies.

Horizon effect on the certain-standard and standard bandits

The frequency of picking the certain-standard bandit was increased in the long versus short horizon (pilot data: t(60)=9.825, p<.001, 95%CI_M=[5.653,8.544], d=-1.258, 95%CI_ES=[-1.605,-0.926]; Fig. Supplementary Figure 17a). Similarly, the frequency of picking the standard bandit was increased in the long horizon (pilot data: t(60)=5.489, p<.001, 95%CI_M=[1.881,4.037], d=-0.703, 95%CI_ES=[-0.989,-0.423]; Fig. Supplementary Figure 17b).

Block and trial effect on the high-value bandit

In addition to the horizon condition, when adding block as a within participant-factor in the repeated-measures ANOVA, there was an additional effect of block on the high-value bandit (pilot data: block main effect: F(2.64, 158.24)=9.062, p<.001, η^2=.131; Horizon main effect: F(1, 60)=71.41, p<.001, η^2=.543; Block-by-horizon interaction effect: F(3, 180)=1.264, p=.288, η^2=.021). This was driven by a decreased selection frequency of the high-value bandit with increasing blocks, mainly due to an decrease from block 1 to block 2 (pairwise comparison: block 1 vs block 2: t(122)=3.261, p=.001, d=.295, 95%CI=[.39, 1.594]; block 1 vs block 3: t(122)=3.991, p<.001, d=.361, 95%CI=[.651, 1.931]; block 1 vs block 4: t(122)=5.184, p<.001, d=.469, 95%CI=[1.051, 2.35]; block 2 vs block 3: t(122)=1.218, p=.226, d=.11, 95%CI=[-0.187, 0.786]; block 2 vs block 4: t(122)=[2.511, p=.013], d=.227, 95%CI=[.15, 1.268]; block 3 vs block 4: t(122)=1.474, p=.143, d=.133, 95%CI=[-0.14, 0.96]). Similarly, when analysing the frequency of picking the high-value bandit per trial, a decrease was observed in the short horizon (linear regression slope vs null slope: t(60)=-4.38, p<.001) and in the long horizon (t(60)=-5.04, p<.001).

No block nor trial effect on the low-value bandit

In addition to the horizon condition, when adding block as a within participant-factor in the repeated-measures ANOVA, there was no observed effect of block on the low-value bandit (pilot data: block main effect: F(2.03, 121.54)=2.219, p=.112, η^2=.036; Horizon main effect: F(1, 60)=11.872, p=.001, η^2=.165; Block-by-horizon interaction effect: F(3, 180)=1.04, p=.376, η^2=.017). Similarly when analysing the frequency of picking the low-value bandit per trial, no evidence of changes across time were observed, neither in the short horizon (linear regression slope vs null slope: t(60)=-1.71, p=.092) nor in the long horizon (t(60)=-1.47, p=.147).

Block and trial effect on the novelty bandit

In addition to the horizon condition, when adding block as a within participant-factor in the repeated-measures ANOVA, there was an additional effect of block on the novelty bandit (pilot data: block main effect: F(2.45, 147.22)=22.947, p<.001, η^2=.277; Horizon main effect: F(1, 60)=73.72, p<.001, η^2=.551; Block-by-horizon interaction effect: F(2.68, 160.77)=.644, p=.571, η^2=.011). This was driven by an increased selection frequency of the novel bandit with increasing blocks (pairwise comparison: block 1 vs block 2: t(122)=-5.072, p<.001, d=.459, 95%CI=[-2.08, -0.912]; block 1 vs block 3: t(122)=-6.427, p<.001, d=.582, 95%CI=[-2.546, -1.347]; block 1 vs block 4: t(122)=-8.157, p<.001, d=.738, 95%CI=[-3.178, -1.937]; block 2 vs block 3: t(122)=-2.093, p=.038, d=.19, 95%CI=[-0.877, -0.024]; block 2 vs block 4: t(122)=-4.001, p<.001, d=.362, 95%CI=[-1.587, -0.536]; block 3 vs block 4: t(122)=-2.316, p=.022, d=.21, 95%CI=[-1.133, -0.089]). Similarly when analysing the frequency of picking the novel bandit per trial, an increase was observed in the short horizon (linear regression slope vs null slope: t(60)=5.88, p<.001) and in the long horizon (t(60)=6.66, p<.001).

Additional block-dependent novelty parameter

Given the observed minor change in the novelty bandit frequency across blocks and trials (cf. above), we extended our model comparison with a model comprising a block-dependent quantity of the novelty bonus, which we named η_B. This model performed similarly to our winning model when looking at the average score (Thompson sampling+ϵ+η: BIC: 524.91 (sd: 1.82); Thompson sampling+ϵ+η+η_B: BIC: 524.75 (sd: 1.84); paired-samples t-test: t(61)=.084, p=.933, 95%CI=[-3.746, 4.075]), but less well than our winning model when looking at the best score per participant (Thompson sampling+ϵ+η: Number of participants: 29; Thompson sampling+ϵ+η+η_B: Number of participants: 19). Importantly adding such a parameter did not affect our main parameter of interest ϵ (correlation between ϵ from the Thompson sampling+ϵ+η model and ϵ from the Thompson sampling+ϵ+η+η_B model: short horizon: r=1, p<.001; long horizon: r=.099, p<.001).

No evidence of meta-learning on number of initial samples

We found no evidence that participants prefer the certain-standard bandit over the standard bandit, neither in the short horizon (linear regression between [certain-standard bandit frequency minus standard bandit frequency] and [trial]: slope vs null slope: t(60)=0.516, p=.608) nor in the long horizon (t(60)=-0.794, p=.43). Moreover, we also find no evidence for choosing any 1-sample bandit (standard or low-value bandit) less compared to the 3-sample bandit over time, neither in the short horizon (linear regression between [3-sample bandit frequency minus 1-sample bandit frequency] and [trial]: slope vs null slope: t(60)=0.85, p=.399), nor in the long horizon (t(60)=-0.326, p=.746).

No difference in bandit colour occurrence

When performing an ANOVA with the participant identifier colour (8 sets of 3 different colours, 8^3 = 24 different colours) and the within factor bandit there was no evidence of a difference in bandit occurrence for each colour (bandit main effect: F(2.34,53.91)=0, p=1, pes=0), meaning that each type of bandit is shown in every combination equally often.

Supplementary tables and figures pilot experiment

**Bandit combination**	N_{trials} **where:**			**Statistics: (μ_{CS} > μ_S) vs (μ_S> μ_{CS})**
	μ_{CS} > μ_S	μ_S> μ_{CS}	μ_{CS} = μ_S	
[Certain-standard, standard, novel]	23.7 (5.22)	24.4 (4.87)	1.84 (2.01)	t(60)=-0.543, p=0.589, 95%CI=[-3.22, 1.85]
[Certain-standard, standard, low-value]	23.3 (4.76)	22.9 (3.8)	3.8 (2.96)	t(60)=0.475, p=0.637, 95%CI=[-1.58, 2.56]

Supplementary Table 19. High-value bandit information (pilot data). Percentage of trials where the certain-standard bandits' 3 initial samples is larger than the initial sample of the standard bandit (μ_{CS} > μ_S) and vice versa (μ_S> μ_{CS}), in the conditions were both bandits are present. Statistics demonstrate that they occur equally often.

Measure	Shapiro normality assumption	Difference of the mean (t-test)	Estimated sample size	Wilcoxon signed-rank test	Estimated sample size
High-value bandit	SH: p=.075<br>LH: p= .539	t(60)=8.45, p<.001,<br>95%CI=[6.92, 11.211],<br>d=1.082,<br>95%CI=[.769, 1.407]	14	V=1737.5,<br>p<.001,<br>r=.779	25
Low-value bandit	SH: p<.001<br>LH: p<.001	t(60)=-3.446, p=.001,<br>95%CI=[-1.568, -.416],<br>d=.441,<br>95%CI=[.178, .708]	69	V=447,<br>p=.002,<br>r=.410	83
Novel bandit	SH: p=.97<br>LH: p=.39	t(60)=-8.586, p<.001,<br>95%CI=[-11.178, -6.954],<br>d=1.099,<br>95%CI=[.784, 1.427]	13	V=83.5,<br>p<.001,<br>r=.786	25
Score 1st SH vs 1st LH	SH: p=.615<br>LH: p=.203	t(60)=6.522, p<.001,<br>95%CI=[.059, .112],<br>d=.835,<br>95%CI=[.545, 1.134]	21	V=1655,<br>p<.001,<br>r=.653	34
Score 1st SH vs all LH	SH: p=.270<br>LH: p=.203	t(60)=-16.096, p<.001,<br>95%CI=[-.245, -.191],<br>d=2.061,<br>95%CI=[1.626, 2.524]	6	V=6,<br>p<.001,<br>r=.864	21
BIC model selection: Thompson+$\epsilon+\eta$ vs Thompson	With ϵ, η: p=.398<br>Without: p=.054	t(60)=-10.187, p<.001,<br>95%CI=[-72.866,-48.946],<br>d=1.304,<br>95%CI=[.967,1.657]	10	V=30,<br>p<.001,<br>r=.842	22
ϵ-greedy parameter	SH: p<.001<br>LH: p<.001	t(60)=-3.23, p=.002,<br>95%CI=[-.058, -.014],<br>d=.413,<br>95%CI=[.152, .679]	79	V=460,<br>p<.001,<br>r=.446	71
Novelty bonus η	SH: p=.004<br>LH: p=.696	t(60)=-9.43, p<.001,<br>95%CI=[-1.265, -.822],<br>d=1.207,<br>95%CI=[.881, 1.548]	12	V=55,<br>p<.001,<br>r=.819	23

Supplementary Table 20. Behavioural and computational modeling summary statistics (pilot data; N=61). Detailed measures of Analysis Step 1. 1st column: the measures we will analyse to verify our hypothesis (cf. Table 1); 2nd column: Shapiro normality test. p<.05 indicates that the normality assumption is violated. 3rd column: t-test statistics of the difference of the means (t, p-value and 95% confidence interval) as well as effect size (Cohen's d and it's 95% confidence interval). 4th column: estimated minimal sample size according to the t-test effect size to reach a 95% power at a significance level of $\alpha = .05$. 5th column: Wilcoxon signed-rank test of the difference of the means and its effect size (r). 6th column: estimated minimal sample size according to the Wilcoxon signed-rank test effect size to reach a 95% power at a significance level of $\alpha = .05$. Sample size estimations were computed in G*Power[10]. SH: Short horizon condition, LH: Long horizon condition.

		Certain-standard		Standard		Novel bandit	Low-value bandit
	Bandit combination	All	When is High-value	All	When is High-value		
Initial samples	[Certain-standard, standard, novel]	5.48 (0.3) [3,10]	5.59 (0.45) [3,10]	5.6 (0.49) [3,10]	6.95 (0.49) [4,10]	-	-
	[Certain-standard, standard, low-value]	5.51 (0.26) [3,10]	5.6 (0.38) [3,10]	5.57 (0.34) [3,10]	6.98 (0.39) [4,10]	-	3.04 (0.22) [2,8]
	[Certain-standard, novel, low-value]	5.57 (0.34) [3,10]		-		-	3.21 (0.29) [2,8]
	[Standard, novel, low-value]	-		5.7 (0.34) [3,10]		-	3.39 (0.28) [2,8]
Short horizon 1st draw	[Certain-standard, standard, novel]	6.41 (0.62) [5.28,8.29]	6.42 (0.73) [4.8,9]	7.07 (0.85) [4.92,9]	7.38 (0.72) [6.14,9]	5.23 (0.74) [3,8]	-
	[Certain-standard, standard, low-value]	5.62 (0.36) [4.83,6.44]	5.6 (0.41) [4.87,6.69]	6.56 (0.57) [4.94,8.12]	6.84 (0.5) [5.88,8.12]	-	3.03 (0.66) [2,5]
	[Certain-standard, novel, low-value]	6.44 (0.57) [5.21,8.43]		-		5.27 (0.58) [3,6.52]	3.2 (1.1) [2,7]
	[Standard, novel, low-value]	-		7.04 (0.78) [5.09,9]		5.33 (0.61) [4.14,8]	3.63 (1.3) [2,8]
Long horizon 1st draw	[Certain-standard, standard, novel]	6.53 (0.91) [4,9]	6.56 (0.97) [4,9]	7.03 (1.1) [4.4,10]	7.44 (0.97) [5.67,10]	5.29 (0.55) [3.78,6.12]	-
	[Certain-standard, standard, low-value]	5.63 (0.39) [5,6.48]	5.62 (0.43) [4.84,6.61]	6.5 (0.49) [5.69,7.88]	6.85 (0.5) [6,8.19]	-	3.23 (1) [2,7.5]
	[Certain-standard, novel, low-value]	6.57 (0.73) [5.24,10]		-		5.44 (0.52) [4.32,6.75]	3.24 (0.98) [2,6]
	[Standard, novel, low-value]	-		7.11 (0.93) [4.85,9.67]		5.42 (0.56) [3.95,8]	3.31 (1.2) [2,10]
Long horizon later draws	[Certain-standard, standard, novel]	5.98 (0.58) [4.54,7.17]	5.96 (0.66) [4.27,7.44]	6.89 (0.75) [4.89,9]	7.17 (0.67) [5.44,9]	6.25 (0.74) [3,8.25]	-
	[Certain-standard, standard, low-value]	5.6 (0.39) [4.74,6.63]	5.6 (0.42) [4.76,6.57]	6.61 (0.5) [5.14,8.2]	6.9 (0.5) [5.73,8.53]	-	3.12 (0.96) [2,7]
	[Certain-standard, novel, low-value]	6.1 (0.49) [4.57,7.08]		-		6.21 (0.73) [4.33,8.06]	3.13 (1) [2,7]
	[Standard, novel, low-value]	-		6.53 (0.59) [5.11,7.91]		5.92 (0.65) [3,7.56]	3.16 (1.1) [2,8]

Supplementary Table 21. Additional reward information (pilot data; N=61). Further analysis of the initial samples (1st row; cf. Supplementary Figure 14a-b), further breakdown of the reward associated to the short horizon sample, the long horizon 1st sample, and the average long horizon sample (2nd-4th row; cf. Supplementary Figure 14c). The rewards are broken down according to which 3 out of 4 bandits were shown on these trials, and according to which bandit was the high-value bandit (highest mean; cf. Supplementary Table 22). Data is given as mean (std) [range].

Bandit combination	N_{trials} where:			Statistics: (μ_{CS} > μ_S) vs (μ_S> μ_{CS})
	μ_{CS} > μ_S	μ_S> μ_{CS}	μ_{CS} = μ_S	
[Certain-standard, standard, novel]	23.7 (5.22)	24.4 (4.87)	1.84 (2.01)	t(60)=-0.543, p=0.589, 95%CI=[-3.22, 1.85]
[Certain-standard, standard, low-value]	23.3 (4.76)	22.9 (3.8)	3.8 (2.96)	t(60)=0.475, p=0.637, 95%CI=[-1.58, 2.56]

Supplementary Table 22. High-value bandit information (pilot data; N=61). Percentage of trials where the certain-standard bandits' 3 initial samples is larger than the initial sample of the standard bandit (μ_{CS} > μ_S) and vice versa (μ_S> μ_{CS}), in the conditions were both bandits are present. Statistics demonstrate that they occur equally often.

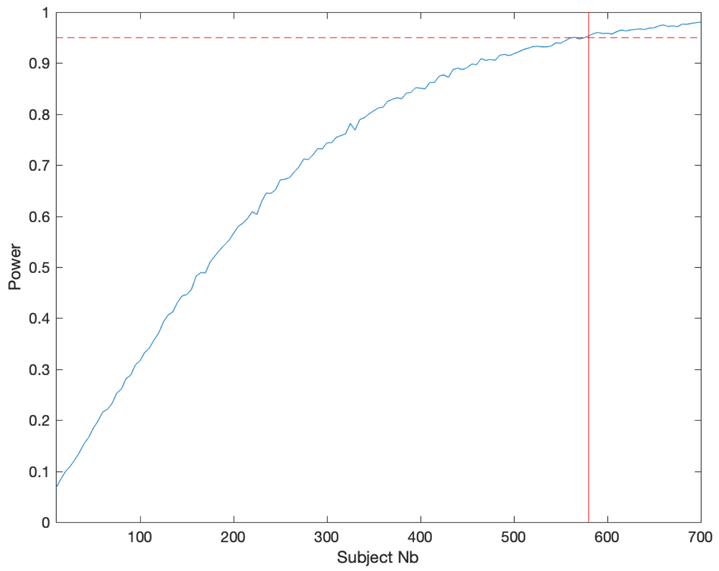

Supplementary Figure 12. Power simulations. Assuming a Pearson correlation of R=0.15 (lowest effect in previous studies[11]) probability of correctly observing a significative correlation (i.e., Power, y-axis) as a function of the sample size (i.e., Participant number, x-axis). The horizontal dashed red line indicates the desired power of 95%. The vertical red line demonstrates that a sample size of N=580 is enough to reach 95% power.

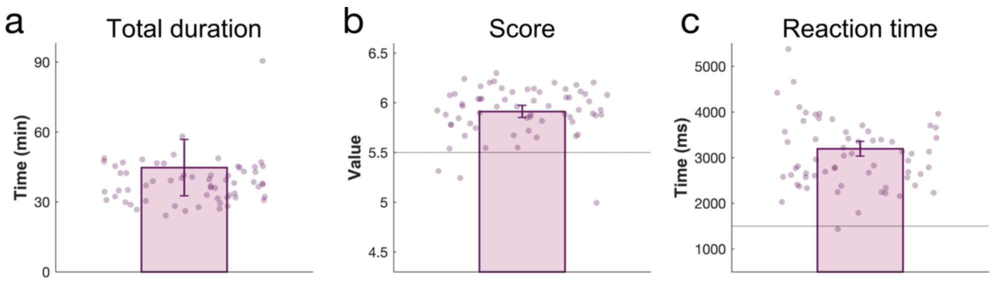

Supplementary Figure 13. Exclusion criteria applied on pilot data (N=64). (a) Total task duration. (b) Participants are excluded if (b) their total score (average apple size) was lower than 5.5 (horizontal line) and if (c) their reaction time on the 1st choice was lower than 1500ms (horizontal line). According to those exclusion criteria, N=3 participants out of the N=64 pilot participants were excluded from further analyses. Data is shown as mean ± 95%CI and each dot represent one participant.

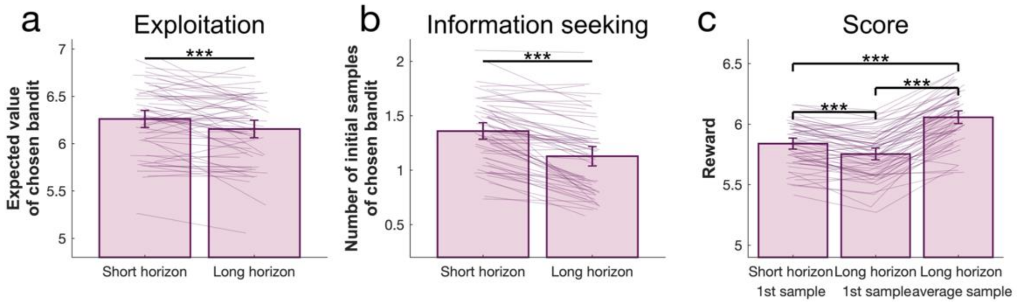

Supplementary Figure 14. Benefits of exploration (pilot data; N=61). Effect of information on performance. (a) The first bandit participants chose as a function of its expected value (average of its initial samples). Participants chose bandits with a lower expected value (i.e., they exploited less) in the long horizon compared to the short horizon. (b) The first bandit participants chose as a function of the number of samples that were initially revealed. Participants chose less known (i.e., more informative) bandits in the long compared to the short horizon. (c) The first draw in the long horizon led to a lower reward than the first draw in the short horizon, indicating that participants sacrificed larger initial outcomes for the benefit of more information. This additional information helped making better decisions in the long run, leading to a higher earning over all draws in the long horizon. *** p<.001. Data are shown as mean ± 95%CI and each dot/line represent one participant.

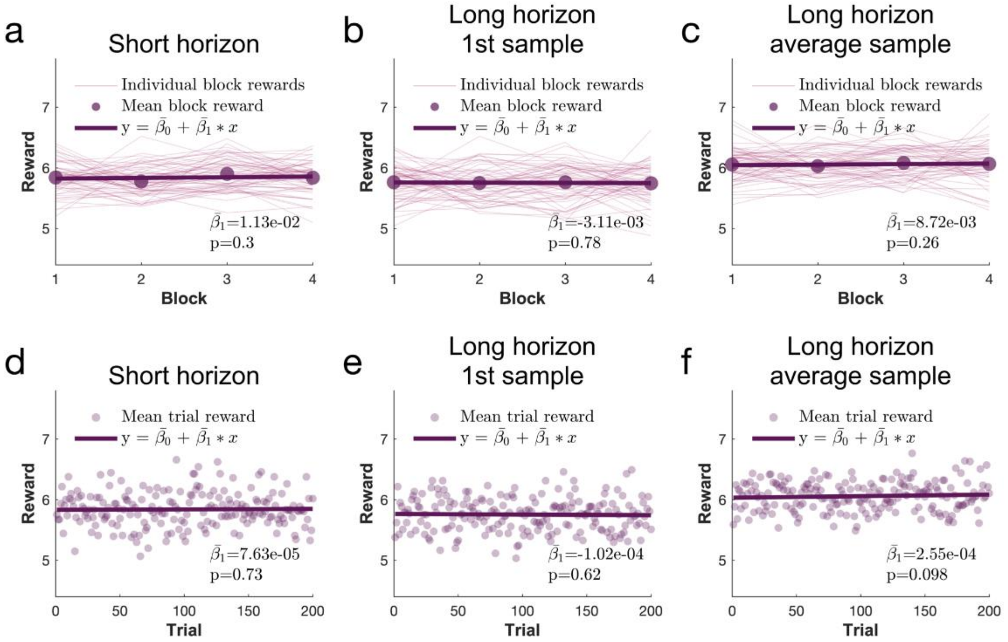

Supplementary Figure 15. Influence of block and trial on score (pilot data; N=61). For each participant, we performed a regression on the average reward per block as well as a regression on the reward per trial. Slopes were compared to a vector of null slopes. When analysing the reward per block: (a) No significant slope was found for the short horizon reward (t(60)=1.036, p=.304, d=.133, 95%CI=[-.0106,.0333]), (b) the long horizons' first sample reward (t(60)=-.286, p=.776, d=-.037, 95%CI=[-.0249,.0187]) nor in (c) the long horizon's average reward (t(60)=1.139, p=.259, d=.146, 95%CI=[-.0066,.024]). Similarly, when analysing the reward per trial: (d) No significant slope was found for the short horizon reward (t(60)=.342, p=.734, d=.044, 95%CI=[-.0004,.0005]), (e) the long horizons' first sample reward (t(60)=-.498, p=.62, d=-.064, 95%CI=[-.0005,.0003]) nor in (f) the long horizon's average reward (t(60)=1.683, p=.098, d=.215, 95%CI=[0,.0006]). There is therefore no evidence that performance changed across time.

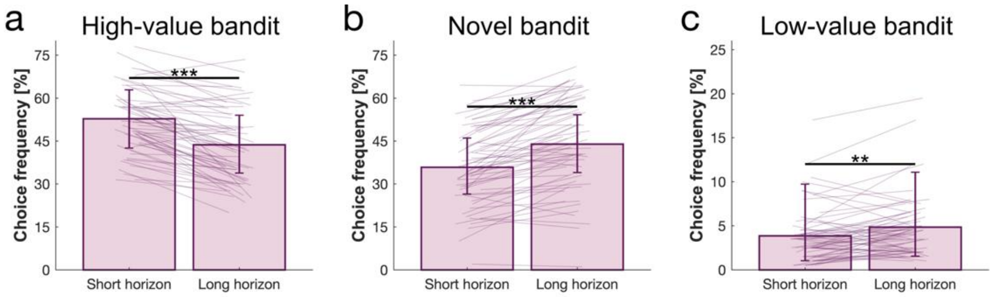

Supplementary Figure 16. Behavioural horizon effects (pilot data; N=61). Choice patterns in the first draw for each horizon condition. Participants sampled from the (a) high-value bandit (i.e., bandit with the highest average reward of initial samples) more in the short horizon compared to the long horizon (indicating reduced exploitation), from the (b) novel bandit more in the long horizon compared to the short horizon (novelty exploration) and from the (c) low-value bandit more in the long horizon compared to the short horizon (value-free random exploration). *** p<.001. Data are shown as mean ± binomial 95%CI and each dot/line represent one participant.

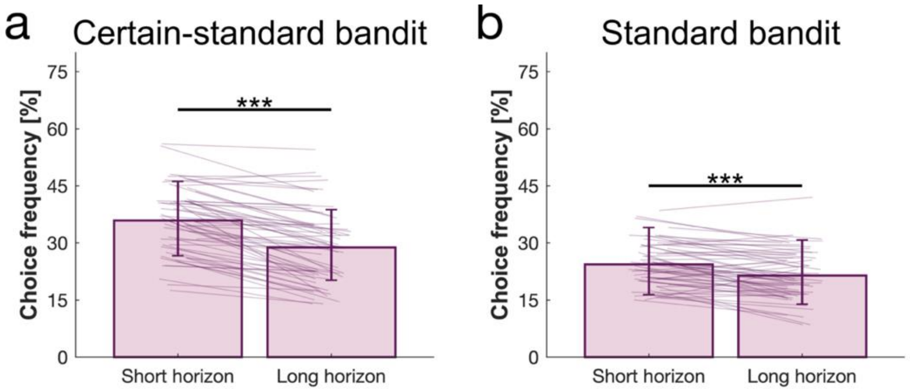

Supplementary Figure 17. Further behavioural horizon effects (pilot data; N=61). Choice patterns in the first draw for each horizon condition. Participants sampled from the (a) certain-standard bandit (i.e., bandit with 3 initial samples) more in the short horizon compared to the long horizon and from the (b) standard bandit (i.e., bandit with 1 initial sample) more in the short horizon compared to the long horizon. *** p<.001. Data are shown as mean ± binomial 95%CI and each dot/line represent one participant.

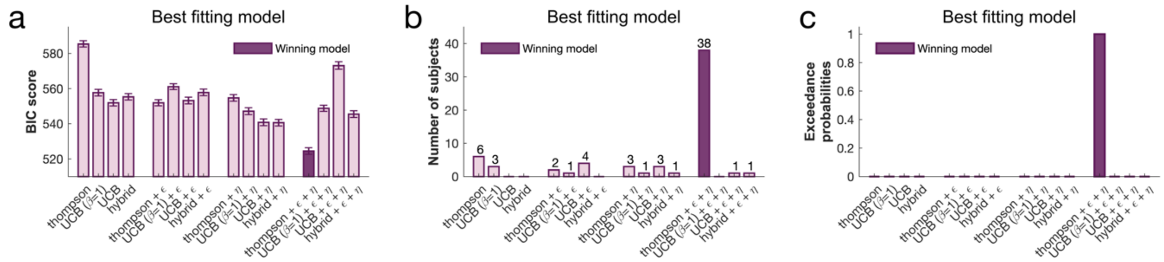

Supplementary Figure 18. Model comparison (pilot data; N=61). Participants use a mixture of exploration strategies. 16 models were compared. Bayesian Information Criterion (BIC) was used for model selection. The Thompson model with both the ϵ-greedy parameter and the novelty bonus η performed best when comparing (a) average BIC score, (b) the number of participants best fitted by each model and (c) the exceedance probabilities when using Bayesian model selection.

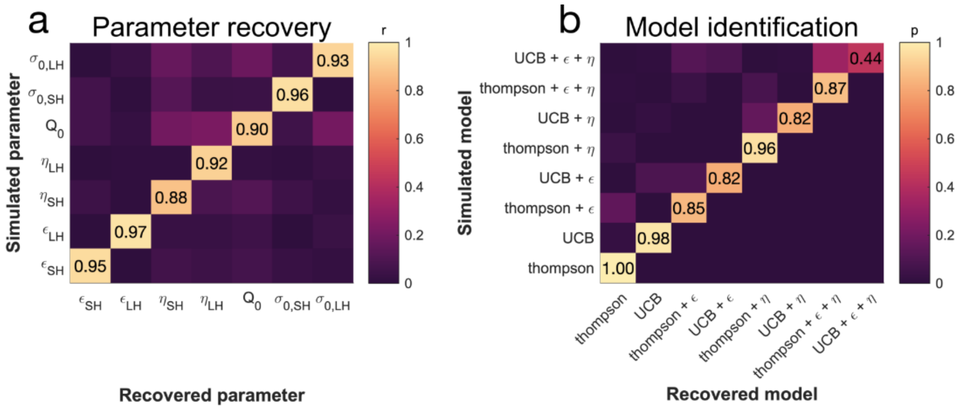

Supplementary Figure 19. Confusion matrices. (a) Parameter recovery. For each parameter of the winning model (Thompson+ η +ϵ from the pilot data), we sampled parameter values from a normal distribution defined by the pilot data mean and standard deviation, which we used to simulate behaviour (for additional simulations cf. Fig. S13). This was performed N=20000 times. For each simulation, we fitted the model and computed the Pearson correlation *r* between the simulated parameters and the fitted parameters. (b) Model identification. For each model, behaviour was simulated N=100 times with parameter values sampled from the pilot data mean and standard deviation. All models were fitted to this simulated data and BIC scores compared. The percentage *p* of how often each fitted model won was computed. The lower recovery of the full-UCB model is likely to reflects the conservative nature of the BIC which punishes its high complexity to the advantage of the simpler (but equally versatile) full-Thompson model.

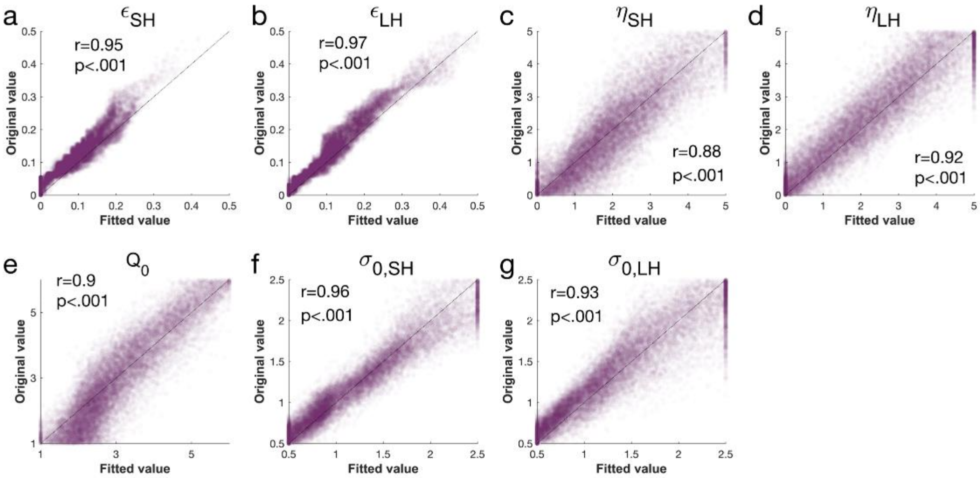

Supplementary Figure 20. Parameter recovery correlations (same data as in Supplementary Figure 21a and Supplementary Figure 6a). For each of the 7 parameters of the winning model, we sampled N=20,000 values from the pilot data fitted mean and variance (for additional simulations cf. Supplementary Figure 24). We simulated behaviour using those parameters, fitted the model and analysed how well the generative parameters (original values) correlated with the recovered ones (fitted parameters). Pearson correlation coefficient = r. Each dot represents one simulation. Source data are provided as a Source Data file.

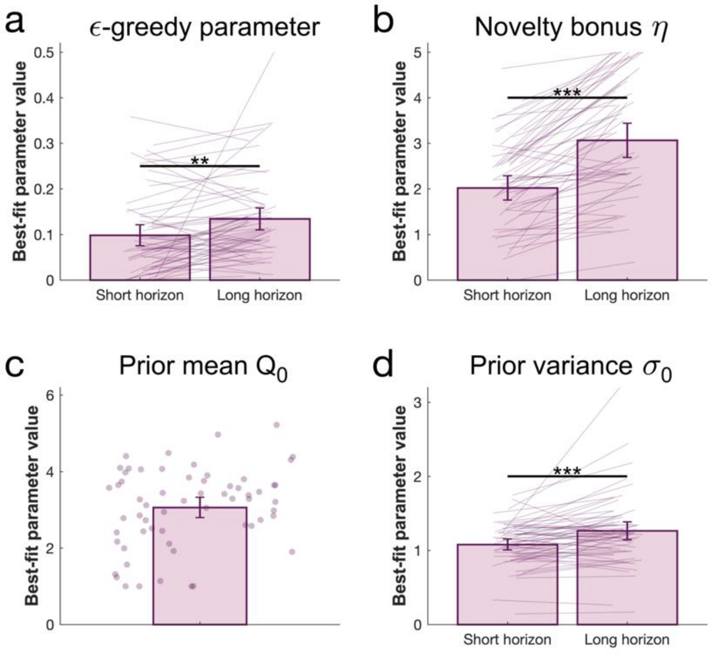

Supplementary Figure 21. Model parameters (pilot data; N=61). The winning model's parameters were fitted to each participant's first draw. (a) Participants had higher values of ϵ (value-free random exploration) in the long compared to the short horizon. (b) They assigned a higher value to novelty (novelty exploration), captured by the novelty bonus η, in the long compared to the short horizon. (c) Participants' beliefs Q_0 about a bandits' mean before seeing any initial samples, and (d) their uncertainty σ_0 about it which was higher in the long compared to the short horizon. ** *p*<.01, *** *p*<.001. Data are shown as mean ± 95%CI and each dot/line represent one participant.

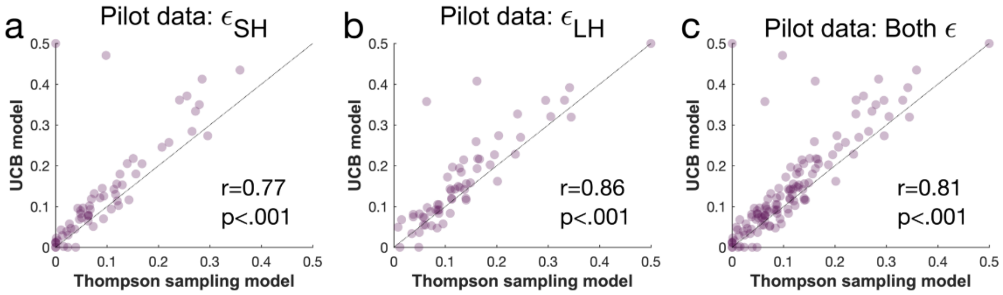

Supplementary Figure 22. Epsilon correlations across complex models (pilot data; N=61). Pearson correlation between ϵ values from the Thompson+ϵ+η model and the UCB+ϵ+η model. (a) Short horizon ϵ. (b) Long horizon ϵ values. (c) Both horizon conditions concatenated.

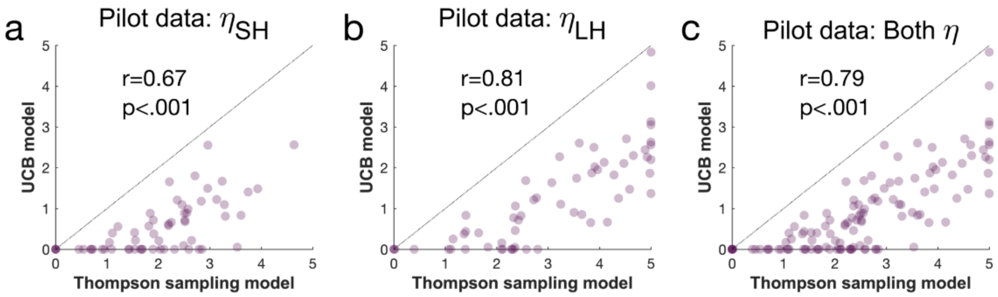

Supplementary Figure 23. Eta correlations (pilot data; N=61). Pearson correlation between η values from the Thompson+ϵ+η model and the UCB+ϵ+η model. (a) Short horizon η. (b) Long horizon η values. (c) Both horizon conditions concatenated.

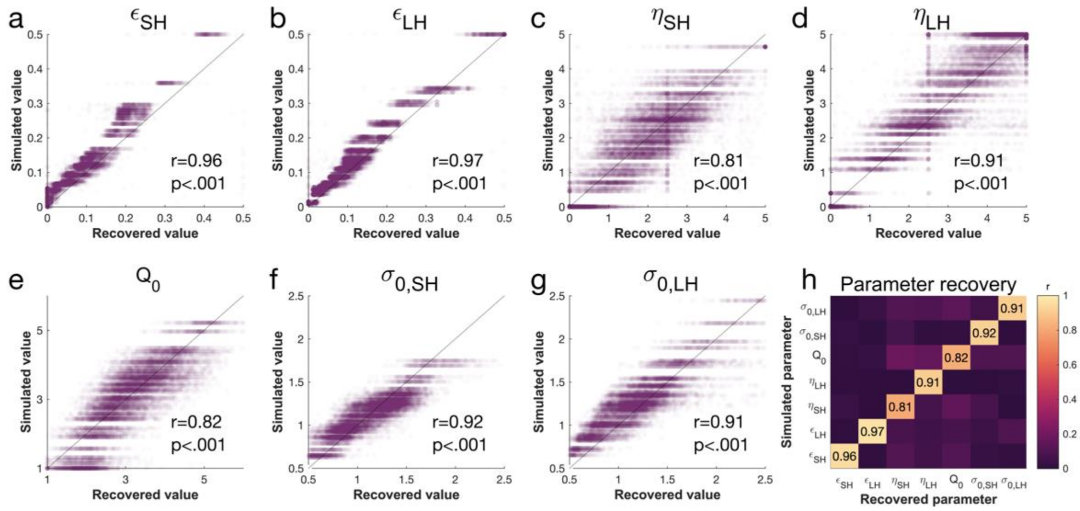

Supplementary Figure 24. Further parameter recovery. For each of the 7 parameters of the winning model, we sampled from the fitted parameter values obtained in the pilot data, which we used to simulate behaviour. This was performed N=20,000 times. For each simulation, we fitted the model and computed the Pearson correlation *r* between the simulated parameters and the fitted parameters. (a-g) Visualisation of the correlation between each parameter and it's respective recovered parameter. (h) Visualisation of all correlations. Pearson correlation coefficient = r. Each dot represents one simulation. Source data are provided as a Source Data file.

Supplementary References

1. Dubois, M. *et al.* Human complex exploration strategies are enriched by noradrenaline-modulated heuristics. *Elife* **10**, 1–34 (2021).
2. Bishop, C. M. Machine Learning and Pattern Recoginiton. in *Information Science and Statistics* (2006).
3. Gershman, S. J. Deconstructing the human algorithms for exploration. *Cognition* **173**, 34–42 (2018).
4. Bhat, H. & Kumar, N. On the derivation of the Bayesian Information Criterion. *Sch. Nat. Sci. Univ. …* (2010).
5. Rigoux, L., Stephan, K. E., Friston, K. J. & Daunizeau, J. Bayesian model selection for group studies - Revisited. *Neuroimage* **84**, 971–985 (2014).
6. Wilson, R. C. & Collins, A. G. E. Ten simple rules for the computational modeling of behavioral data. *Elife* **8**, 1–33 (2019).
7. Allison, C., Auyeung, B. & Baron-Cohen, S. Toward brief 'red flags' for autism screening: The short Autism Spectrum Quotient and the short Quantitative Checklist in 1,000 cases and 3,000 controls. *J. Am. Acad. Child Adolesc. Psychiatry* **51**, 202-212.e7 (2012).
8. Martin, M. M. & Rubin, R. B. A New Measure of Cognitive Flexibility. *Psychol. Rep.* **76**, 623–626 (1995).
9. Condon, D. M. & Revelle, W. The international cognitive ability resource: Development and initial validation of a public-domain measure. *Intelligence* **43**, 52–64 (2014).
10. Faul, F., Erdfelder, E., Lang, A. G. & Buchner, A. G*Power 3: A flexible statistical power analysis program for the social, behavioral, and biomedical sciences. *Behav. Res. Methods* **39**, 175–191 (2007).
11. Rollwage, M., Dolan, R. J. & Fleming, S. M. Metacognitive Failure as a Feature of Those Holding Radical Beliefs. *Curr. Biol.* **28**, 4014-4021.e8 (2018).
