# Supplementary material for: Value-free random exploration is linked to impulsivity
Source: Nat Commun. 2022 Aug 4;13:4542. doi: 10.1038/s41467-022-31918-9 (PMC9352791; doi:10.1038/s41467-022-31918-9)
Supplement: Supplementary file 3 — Reporting Summary [file 41467_2022_31918_MOESM3_ESM.pdf]

nature portfolio

Corresponding author(s): Magda Dubois
Last updated by author(s): Apr 12, 2022

Reporting Summary

Nature Portfolio wishes to improve the reproducibility of the work that we publish. This form provides structure for consistency and transparency in reporting. For further information on Nature Portfolio policies, see our Editorial Policies and the Editorial Policy Checklist.

Statistics

For all statistical analyses, confirm that the following items are present in the figure legend, table legend, main text, or Methods section.

n/a	Confirmed	
☐	☒	The exact sample size (*n*) for each experimental group/condition, given as a discrete number and unit of measurement
☐	☒	A statement on whether measurements were taken from distinct samples or whether the same sample was measured repeatedly
☐	☒	The statistical test(s) used AND whether they are one- or two-sided<br>*Only common tests should be described solely by name; describe more complex techniques in the Methods section.*
☐	☒	A description of all covariates tested
☐	☒	A description of any assumptions or corrections, such as tests of normality and adjustment for multiple comparisons
☐	☒	A full description of the statistical parameters including central tendency (e.g. means) or other basic estimates (e.g. regression coefficient) AND variation (e.g. standard deviation) or associated estimates of uncertainty (e.g. confidence intervals)
☐	☒	For null hypothesis testing, the test statistic (e.g. *F*, *t*, *r*) with confidence intervals, effect sizes, degrees of freedom and *P* value noted<br>*Give P values as exact values whenever suitable.*
☒	☐	For Bayesian analysis, information on the choice of priors and Markov chain Monte Carlo settings
☒	☐	For hierarchical and complex designs, identification of the appropriate level for tests and full reporting of outcomes
☐	☒	Estimates of effect sizes (e.g. Cohen's *d*, Pearson's *r*), indicating how they were calculated

Our web collection on statistics for biologists contains articles on many of the points above.

Software and code

Policy information about availability of computer code

Data collection	Data was collected on the online platform Prolific, with a code coded in ReactJS (version: 16.13.0)
Data analysis	Data was analysed using MATLAB (version: 2016b) and R (version: 3.6.3). Sample sizes estimation were computed using G*Power (version 3.1.9.6). Scripts for the task can be found at: https://github.com/MagDub/MFweb-app, and scripts for data analysis at: https://github.com/MagDub/MFweb-data_analysis.

For manuscripts utilizing custom algorithms or software that are central to the research but not yet described in published literature, software must be made available to editors and reviewers. We strongly encourage code deposition in a community repository (e.g. GitHub). See the Nature Portfolio guidelines for submitting code & software for further information.

Data

Policy information about availability of data

All manuscripts must include a data availability statement. This statement should provide the following information, where applicable:

- Accession codes, unique identifiers, or web links for publicly available datasets
- A description of any restrictions on data availability
- For clinical datasets or third party data, please ensure that the statement adheres to our policy

The raw (anonymized) and processed data are available at https://github.com/MagDub/Mfweb-data (pilot data at: https://github.com/MagDub/Mfweb-pilot_data). The data generated in this study are provided in the Source Data file.

Field-specific reporting

Please select the one below that is the best fit for your research. If you are not sure, read the appropriate sections before making your selection.

☐ Life sciences ☒ Behavioural & social sciences ☐ Ecological, evolutionary & environmental sciences

For a reference copy of the document with all sections, see nature.com/documents/nr-reporting-summary-flat.pdf

Behavioural & social sciences study design

All studies must disclose on these points even when the disclosure is negative.

Study description	In this behavioural online study we looked at individual differences in the exploration-exploitation trade-off.
Research sample	Subjects were recruited through Prolific. To take part in the study, subjects had to be above 18 years of age and have their current residence in the UK. The final dataset consisted of N=580 healthy human subjects (304 female) with a mean age of 35.7 (sd=13.6, range=[18,83]). We believe that this sample is representative. We chose this adult sample because we were not interested in developmental changes in this study.
Sampling strategy	Sample size was estimated in order to reach a 95% power assuming an effect size similar to what has been observed in previous studies and in the pilot data set (sampling procedure = random).
Data collection	Data is collected on the online platform Prolific. Participants registered on this platform and then recieved a link to the behavioural task. The researcher was never besides the participant.
Timing	The data was collected between the 31st of March and the 9th of April 2021.
Data exclusions	N=77 subjects were excluded from the final dataset because they did not fill the (behavioural) inclusion criteria. Exclusion criteria: data was incomplete, the mean score (i.e., apple size) was lower than 5.5 indicating subjects were performing at chance level, the first draw mean reaction time was faster than 1500ms (based on our pilot data and previous study) indicating subjects were not allocating much thought to their choice and if subjects failed at least one attention check during the questionnaires meaning that they were not reading the questions.
Non-participation	No participants dropped out after starting the study.
Randomization	Participants will not be allocated into experimental groups

Reporting for specific materials, systems and methods

We require information from authors about some types of materials, experimental systems and methods used in many studies. Here, indicate whether each material, system or method listed is relevant to your study. If you are not sure if a list item applies to your research, read the appropriate section before selecting a response.

Materials & experimental systems

n/a	Involved in the study	
☒	☐	Antibodies
☒	☐	Eukaryotic cell lines
☒	☐	Palaeontology and archaeology
☒	☐	Animals and other organisms
☐	☒	Human research participants
☒	☐	Clinical data
☒	☐	Dual use research of concern

Methods

n/a	Involved in the study	
☒	☐	ChIP-seq
☒	☐	Flow cytometry
☒	☐	MRI-based neuroimaging

Human research participants

Policy information about studies involving human research participants

Population characteristics	The final dataset consisted of N=580 healthy human subjects (304 female) with a mean age of 35.7 (sd=13.6, range=[18,83]).
Recruitment	Participants were recruited through the online platform Prolific. The sample may be biased because it comprises people playing online tasks, so mainly students or un-employed people.
Ethics oversight	UCL research ethics committee (REC No 15301/001)

Note that full information on the approval of the study protocol must also be provided in the manuscript.
